# Why Some People Live Past 100: The Role of the Immune System in Centenarians, Semi-Supercentenarians and Supercentenarians

**DOI:** 10.3390/ijms27188420

**Published:** 2026-09-21

**Authors:** Calogero Caruso, Giulia Accardi, Anna Aiello, Anna Calabrò, Chiara Puleo, Rosa Zarcone, Giuseppina Candore

**Affiliations:** Laboratory of Immunopathology and Immunosenescence, Department of Biomedicine, Neuroscience, and Advanced Diagnostics, University of Palermo, Corso Tukory 211, 90134 Palermo, Italy; giulia.accardi@unipa.it (G.A.); anna.aiello@unipa.it (A.A.); anna.calabro@unipa.it (A.C.); chiara.puleo@unipa.it (C.P.); rosa.zarcone@unipa.it (R.Z.); giuseppina.candore@unipa.it (G.C.)

**Keywords:** centenarians, immune ageing, immunosenescence, inflammaging, semi-supercentenarians, supercentenarians

## Abstract

Centenarians, semi-supercentenarians and supercentenarians (i.e., oldest centenarians) display complex and heterogeneous immune remodelling. In this setting, immune attrition, memory, cytotoxic defence, inflammatory regulation and tissue surveillance remain sufficiently balanced to support survival with relatively preserved health. This review examines these processes within an integrated framework encompassing sex and gender differences, genetic background, the exposome and immunobiography. We consider how haematopoietic stem cell ageing, age-related myeloid bias, thymic involution, immune ageing and inflammaging contribute to immune-system remodelling across the life course. Findings from conventional phenotyping and single-cell transcriptomic studies in centenarians, semi-supercentenarians and supercentenarians indicate selective immune remodelling rather than preservation of a youthful immune system. These changes include NK-cell expansion, differentiated B-cell states, enrichment of effector-memory CD8^+^ T cells, marked expansion of clonally selected cytotoxic CD4^+^ T cells and increased GZMK^+^GZMB^−^ CD8^+^ T cell populations. Exceptional longevity also appears to depend on limiting the detrimental consequences of chronic inflammation through delayed or better-controlled inflammaging, restrained NLRP3 inflammasome activation and preserved capacity to buffer oxidative stress. Thus, extreme survival appears to reflect successful accommodation of age-related immune change through sustained cytotoxic surveillance, immune memory, inflammatory control and favourable lifelong adaptation to antigenic exposures.

## 1. Introduction

Human longevity is a complex phenotype influenced by a wide range of interacting genetic, physiological, cellular, environmental, geographic behavioural and socioeconomic factors. Geroscience therefore considers longevity as the result of multiple determinants acting across the life course rather than as the consequence of a single biological programme. In addition to inherited predisposition and environmental exposures, stochastic processes also contribute to inter-individual variation in ageing. Random molecular and cellular events, including somatic mutations, epigenetic drift, clonal dynamics and variable responses to biological damage, may progressively generate divergent ageing trajectories even among individuals with broadly similar genetic backgrounds and life environments. The relative contribution of these determinants varies among individuals and populations and changes over time [1,2,3,4,5,6,7].

Sex and gender are important modifiers of ageing trajectories, acting through biological mechanisms as well as through differences in behaviour, social roles, occupational exposures, healthcare use and other components of the exposome. The gut microbiota adds a further layer of complexity, as its composition is shaped by age, diet, medications, environment and lifestyle, while in turn influencing metabolism, immune regulation and systemic inflammation. Thus, exceptional longevity is likely to emerge from the lifelong interaction among genetic background, sex, gender, microbiota, environmental exposures and stochastic biological events, rather than from any single determinant [1,8,9,10].

Within this broad framework, the present review focuses on the contribution of immune ageing to exceptional longevity. The immune system is progressively remodelled across the life course by infections, vaccinations, persistent viruses, nutrition, microbiota, environmental exposures and other individual experiences. These cumulative influences generate a personal immunobiography, which may help explain why centenarians, semi-supercentenarians and supercentenarians display highly heterogeneous immune phenotypes despite sharing exceptional survival. We therefore examine how immune decline, compensatory remodelling, inflammatory regulation, immunological memory and cytotoxic surveillance may combine in different ways to support resilience at the extreme limits of human lifespan [11,12,13].

## 2. Sex, Gender and the Female Exceptional Longevity Advantage

### 2.1. Demographic Features of Exceptional Longevity

Long-living individuals (LLIs; ≥90 years), centenarians (≥100 years), semi-supercentenarians (≥105 years) and supercentenarians (≥110 years) are the subject of intensive scientific investigation because they provide valuable models for exploring the biological, demographic and environmental determinants of exceptional longevity. Italy has one of the highest life expectancies and one of the largest centenarian populations worldwide, making it a particularly informative setting in which to examine the relative distribution of these different longevity categories. As of 1 January 2025, 23,548 centenarians were living in Italy. The population was predominantly female, with women accounting for approximately 83% of the total. Among semi-supercentenarians (*n* = 724), the proportion of women increased to 90.7%. According to the Italian supercentenarian database (accessed 12 September 2026), there were an estimated 20 living supercentenarians, only one of whom was man. Thus, there was approximately one semi-supercentenarian for every 33 centenarians and one supercentenarian for every 1177 centenarians [14,15]. The demographic, clinical and immunological relevance of the main categories of exceptional longevity is summarised in Table 1.

### 2.2. Literature Search Strategy

Although this is a narrative review, we provide a brief description of how the literature base was assembled. The search covered literature published from January 2025 onwards, building on evidence identified through the authors’ more than a decade of previous work in the field, and was subsequently updated through August 2026 to incorporate the most recent publications. PubMed was searched for English-language articles, and the search was supplemented with relevant grey literature from institutional websites. Retrieved publications were screened for relevance to the scope of the review, and the reference lists of key articles, including systematic reviews and relevant guidelines, were manually examined to identify additional eligible studies. The search strategy combined the term “longevity” (in the title or abstract) with the key words “centenarians”, “semi-supercentenarians”, “supercentenarians”, “immunosenescence”, “inflammaging”, “sex”, and “gender”, used individually or in combination.

### 2.3. Biological Mechanisms Underlying the Female Longevity Advantage

The marked female predominance previously reported reflects a broader demographic phenomenon: women live longer than men in almost every region of the world, with a survival advantage of approximately 5–6 years in Europe. This raises an obvious question: why do women live longer than men? The explanation is complex and involves both biological factors related to sex, including genetics, anatomy and sex hormones, and sociocultural factors related to gender, such as occupational and recreational activities, social expectations, risk-taking behaviours and lifestyle.

A comparative analysis of several hundred mammalian and avian species provides an informative evolutionary perspective. Among mammals, adult females live approximately 12% longer than males, whereas the opposite pattern is observed in birds, in which adult males live approximately 5% longer than females. This finding is consistent with the heterogametic sex hypothesis, according to which the sex carrying two copies of the same sex chromosome has a survival advantage. In mammals, females are XX and males are XY, whereas in birds, males are ZZ and females are ZW. In female mammals, this survival advantage may be partly related to random X-chromosome inactivation (XCI), which occurs early in development to ensure dosage compensation. This process generates cellular mosaicism: when one X chromosome carries a deleterious allele, approximately half of the cells may still express the healthy allele from the other X chromosome. Such mosaicism may therefore mitigate the phenotypic consequences of harmful X-linked variants [30].

XCI is, however, incomplete. Up to one-third of X-linked genes may escape inactivation and be expressed from both the active and inactive X chromosomes in female cells, although the extent of this escape varies between genes and individuals. This is particularly relevant to immunity because several genes involved in immune responses are located on the X chromosome. Their greater expression in females may contribute to stronger immune protection, while also increasing susceptibility to many autoimmune diseases. Overall, this enhanced immune responsiveness may nevertheless confer a survival advantage [31,32].

**Figure 1:**
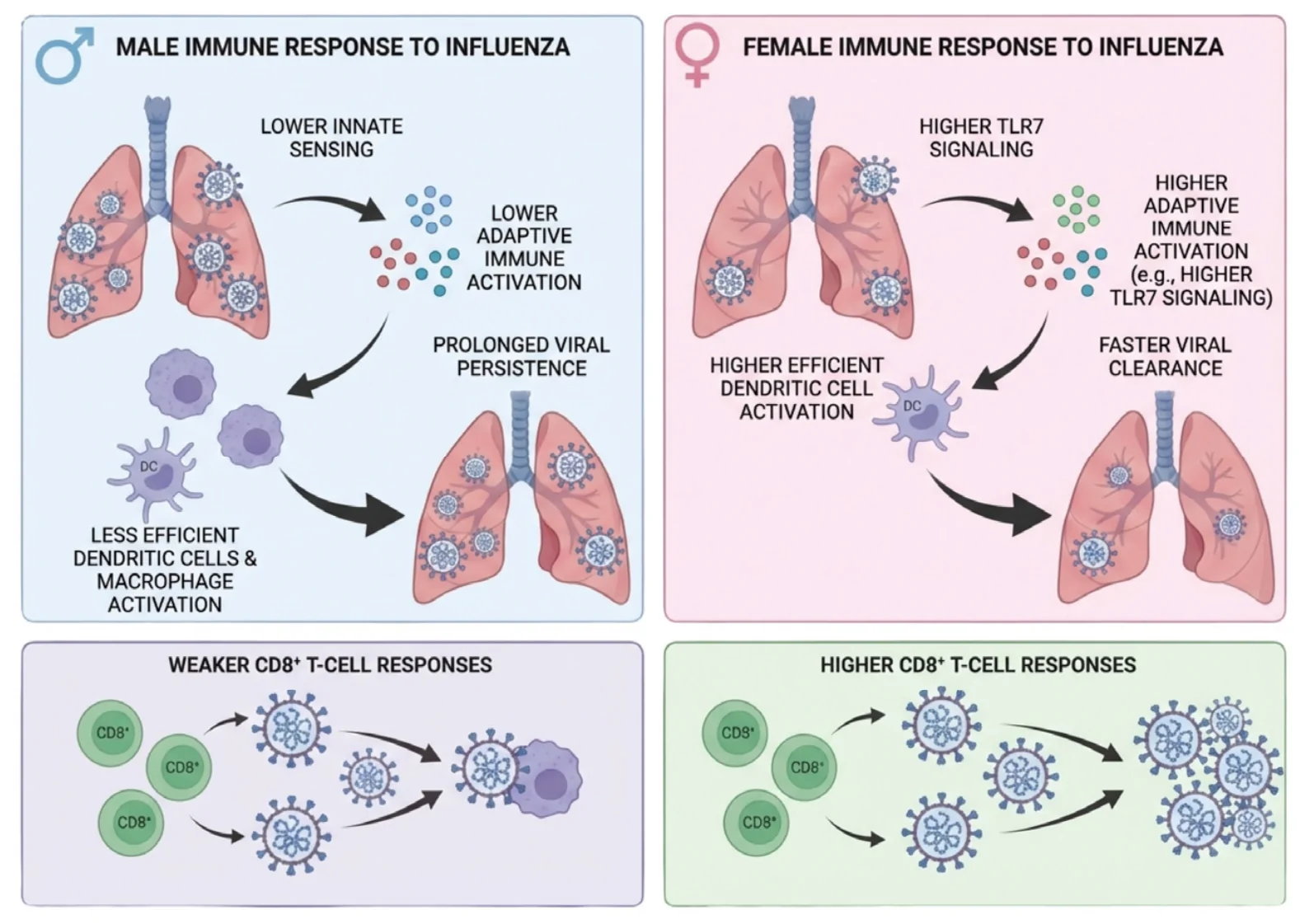
**Sex-associated immunological mechanisms contributing to stronger antiviral responses to influenza in females than in males.** The figure summarises key pathways involved in viral sensing and antiviral immunity. In females, increased TLR7 gene dosage, due to escape from X-chromosome inactivation and consequent biallelic expression, may enhance TLR7-mediated viral recognition and promote greater interferon production by DCs. These sex-associated differences in DC function can, in turn, contribute to more effective adaptive immune responses [36], illustrated here by increased CD8^+^ T cell activity [37]. TLR, toll-like receptor; DCs, dendritic cells. Created with BioRender. Calabrò, A. (2026) https://BioRender.com/eqazsoy. Published from [33] under a Creative Commons CC-BY license.

These mechanisms are well illustrated by recent reviews of sex-related differences in the immune response to influenza viruses and SARS-CoV-2 [33,34]. Antiviral responses may be stronger in females partly because of the higher functional dosage of toll-like receptor (TLR)7, an X-linked gene that may be expressed from both X chromosomes in dendritic cells. This can result in greater interferon (IFN) production, which in turn promotes a more effective adaptive immune response, represented in the figure by enhanced CD8^+^ T cell activity [32,35] (Figure 1).

Figure 1 illustrates some of these mechanisms in the context of influenza infection.

A recent ex vivo human study further showed that post-pubertal cisgender women have higher numbers of class-switched memory B cells than age-matched men. This difference emerges only after puberty and disappears after menopause, suggesting a major contribution of sex hormones. B cells express high levels of the oestrogen receptor ESR2, which is associated with genes involved in class-switch recombination. In transgender men with an XX chromosomal complement, oestrogen suppression reduced the number of these memory B cells, whereas oestrogen administration in transgender women with an XY chromosomal complement did not increase them. Similarly, postmenopausal women receiving hormone replacement therapy had higher levels of these cells than women not receiving such treatment. These findings suggest that sex hormones and sex chromosomes interact, with oestrogen affecting this B cell population primarily in individuals with an XX chromosomal background [38].

### 2.4. Gender, Exposome and Survival Differences

Socially shaped behaviours and environmental exposures also contribute to differences in survival between men and women. Women from older generations have generally been less likely than men to smoke heavily, misuse alcohol or engage in hazardous activities, and have often shown greater attention to disease prevention, healthcare use and self-care. Men, by contrast, have more frequently engaged in risk-taking behaviours, including dangerous driving, excessive alcohol consumption and interpersonal violence, all of which may contribute to higher mortality. These patterns should be interpreted primarily as gender-related differences because they reflect social expectations, cultural norms, occupational roles and learned behaviours rather than biological sex alone. Gender also influences the exposome, broadly understood as the cumulative set of environmental, occupational, behavioural and social exposures experienced throughout life. Differences in employment, caregiving responsibilities, socioeconomic circumstances, diet, physical activity, smoking, alcohol consumption, psychosocial stress, access to healthcare and exposure to environmental pollutants may generate distinct lifetime exposure profiles in men and women. These gender-related exposomic differences may interact with biological sex to influence immune function, disease susceptibility, ageing trajectories and survival. Gender differences are also apparent in attitudes towards diseases, participation in preventive programmes, willingness to seek medical advice and the social responsibilities traditionally assigned to men and women. Greater women longevity is therefore unlikely to have a single explanation. Rather, it probably reflects the interaction between biological advantages and anthropological, cultural, social and exposomic factors [10,33,34].

These patterns are neither universal nor immutable. As lifestyles, social roles and environmental exposures become increasingly similar between men and women, younger women may adopt behaviours historically more common among men, including smoking, harmful alcohol consumption and other forms of risk-taking. The magnitude of the women survival advantage may therefore change in future generations.

## 3. Genetics, Exposome and Immunobiography in Exceptional Longevity

### 3.1. Genetic Contribution to Exceptional Longevity

From an evolutionary perspective, exceptional longevity may be interpreted as the capacity to preserve effective biological repair and homeostasis beyond reproductive life. Ageing does not arise from a single deterministic programme, but from the progressive accumulation of molecular and cellular damage that eventually exceeds the capacity of maintenance and repair mechanisms. Longevity-associated genes should therefore not be regarded as direct determinants of longevity, but rather as variants that enhance resistance to damage, disease and functional decline [39,40,41,42].

Family and twin studies support a genetic contribution to human longevity. Brothers of centenarians have shown a survival advantage over brothers-in-law who were likely to have shared broadly similar adult living conditions, indicating that familial longevity cannot be explained by environmental factors alone. This difference was less evident between sisters and sisters-in-law, suggesting that inherited factors may exert a stronger influence on exceptional survival in men than in women [43]. Twin studies estimate that genetics accounts for approximately 25% of the overall variability in lifespan, with its contribution increasing at extreme ages and possibly being more pronounced in men [44,45,46].

The relative influence of genes and environment also changes across the life course. Up to approximately the eighth decade, diet, physical activity, smoking, socioeconomic circumstances and access to healthcare are generally stronger determinants of health and survival. At more advanced ages, however, genetic factors may become increasingly important in supporting resilience and delaying the onset of age-related diseases. Among centenarians, their estimated contribution may reach approximately 33% in women and 48% in men. Nevertheless, the effects of individual variants are generally modest, suggesting that longevity is a highly polygenic and multifactorial trait [46,47,48,49,50].

Genetic background interacts continuously with environmental exposures throughout life [51]. This gene–environment interplay may partly explain why genome-wide association studies (GWAS) have identified relatively few common variants that are consistently associated with longevity, particularly when genetically, culturally and environmentally heterogeneous populations are analysed together. Overall, longevity-associated variants appear to act mainly by protecting against age-related diseases, especially cardiovascular disease, which remains the leading cause of death worldwide and accounts for an estimated 17.9 million deaths annually [52]. Accordingly, Garagnani et al. [53] performed high-coverage whole-genome sequencing in 81 Italian semi-supercentenarians and supercentenarians aged 105 years or older (mean age 106.6 ± 1.6 years), together with 36 healthy, unrelated, geographically matched controls (mean age 68.0 ± 5.9 years). Their findings suggest that individuals with extreme longevity carry a distinctive genetic profile linked to more effective maintenance of genomic integrity. This was supported by the distribution of both common and rare germline variants and by the observation of a lower somatic mutational burden than in the younger control group. Overall, the study pointed to DNA repair pathways as important contributors to healthy ageing and to protection against cardiovascular disease in exceptionally long-lived individuals.

As previously stated, GWAS conducted in centenarian populations have identified only a limited number of variants reaching genome-wide statistical significance. This is consistent with the view that exceptional longevity is a highly complex trait, shaped by the combined influence of multiple genetic variants, each exerting a relatively small individual effect but collectively contributing substantially to survival [42]. Importantly, the genetic contribution to longevity appears to become progressively stronger among individuals who survive beyond 100 years at the most extreme ages [54].

Contrary to earlier assumptions, several studies have also shown that centenarians carry many disease-associated genetic variants that are common in the general population. This observation suggests that exceptional longevity may depend not simply on the absence of deleterious variants, but also on the presence of protective genetic factors that buffer their effects. These protective mechanisms may contribute to slowing biological ageing, reducing vulnerability to age-related diseases, and limiting the impact of conditions that usually promote premature mortality [54].

### 3.2. Gene–Environment Interactions and the Exposome

Genetics is important, but its effects are strongly influenced by environmental and life-course exposures. This is illustrated by Gurinovich et al., [55] who found no association of APOE ε2 with longevity or APOE ε4 with Alzheimer’s disease in southern Italy. However, both associations were observed in centenarians of southern Italian ancestry born or raised in the United States. This difference may reflect contrasting environments. Centenarians raised in southern Italy were often exposed to a low-calorie Mediterranean diet, rural living, limited pollution, traditional food systems, natural daily rhythms and strong social networks. Those raised in the United States experienced greater urbanisation, different diets, educational and socioeconomic conditions, and distinct exposure to infections and pollutants. Thus, populations with a similar genetic background may follow different ageing trajectories depending on their environment [5,6,55].

In this context, the exposome, the totality of external and internal exposures accumulated throughout life, becomes particularly relevant. It includes the general external environment, such as urban or rural residence, climate, socioeconomic status and education; the specific external environment, including diet, physical activity, smoking, occupational exposures, infections and individual behaviours; and the internal environment, comprising metabolism, gut microbiota, inflammatory responses, oxidative stress and ageing itself [5,6].

In addition to what was discussed above, these findings may also help explain why GWAS identify relatively few robust longevity-associated variants when different populations are pooled. Although such analyses increase statistical power, they may obscure the ecological setting in which exceptional longevity develops [42,56].

Studies of month or season of birth further emphasise the contribution of the perinatal exposome. Seasonal patterns may reflect differences in maternal nutrition, infectious exposure, temperature, socioeconomic conditions and other environmental influences acting around conception, during pregnancy and in early infancy. The fact that the most favourable birth months vary between populations reinforces this interpretation, because the biological meaning of season of birth depends on the specific climatic, nutritional, epidemiological and historical context. These observations suggest that early-life exposures may leave a lasting biological imprint that influences the probability of surviving to extreme ages [57,58].

### 3.3. Immunobiography and Personalised Immune Ageing

If the genetics of longevity is ecologically dependent, this principle applies even more strongly to the immune system. The ageing of the immune system is shaped not only by inherited factors, but also by the cumulative effects of infections, vaccinations, nutrition, microbiota, environmental exposures, psychosocial stress and other events encountered throughout life. This individual immunological history has been termed immunobiography [11]. Consequently, the immunological features observed in LLIs should not necessarily be expected to occur in every population or in every centenarian. Different immunobiographies may lead to distinct immune trajectories and to multiple, equally successful patterns of adaptation compatible with exceptional longevity.

## 4. Haematopoietic and Thymic Function in Ageing and Longevity

### 4.1. Haematopoietic Stem-Cell Ageing and Myeloid Bias

Older adults and centenarians showed a tendency towards reduced absolute counts of CD34^+^, hematopoietic stem and progenitor cells. This difference became statistically significant when the two older groups were combined and compared with young adults, whereas no further reduction was observed between old age and exceptional longevity. Despite their lower numbers, CD34^+^ cells retained responsiveness to haematopoietic cytokines and preserved their ability to generate myeloid colonies [59]. CD34 is commonly used as a surface marker to enrich for human haematopoietic stem cells (HSCs) and progenitor cells, but it does not identify bona fide HSCs by itself, as the CD34^+^ compartment also includes more committed progenitor populations.

HSCs are increasingly viewed as central regulators of the ageing of the immune system and, potentially, of longevity, because they generate all circulating immune-cell lineages and thereby influence immune function throughout the organism. With advancing age, HSCs undergo molecular, epigenetic and microenvironmental changes that alter their differentiation potential. One major consequence is an age-related myeloid bias, in which haematopoiesis progressively shifts away from lymphoid output and towards the production of myeloid cells [60]. This imbalance contributes to the decline of adaptive immunity, reduced generation of B and T lymphocytes, impaired vaccine responses and increased susceptibility to infection, while favouring the expansion of innate immune populations that can sustain inflammaging, promoting age-related inflammatory diseases. This HSC-driven remodelling provides a plausible mechanistic link between immunosenescence and late-life diseases. Ageing HSCs do not simply produce fewer immune cells; rather, they generate a qualitatively altered immune system, characterised by a myeloid-biased haematopoiesis at the expense of lymphoid cell production. The resulting reduction in lymphoid competence limits immune diversity and adaptive responses, whereas excess myeloid differentiation may amplify inflammatory tone and contribute to tissue damage [61].

Experimental studies suggest that correction of age-related HSC defects may have systemic effects relevant to healthy ageing. The activity of the small Rho GTPase cell division cycle 42 (Cdc42) is increased in the blood of older humans and in several tissues of aged mice. It acts as a molecular switch, cycling between an inactive GDP-bound state and an active GTP-bound state. Constitutive activation of Cdc42 has been shown to induce features of premature ageing and to shorten lifespan in mice. This hyperactivation, indeed, is associated with loss of cell polarity, epigenetic alterations, and reduced regenerative capacity, thereby contributing to the aged HSC phenotype [62]. In earlier work, Florian et al. [62] reported that a brief 16 h ex vivo exposure of aged HSCs to a Cdc42 activity-specific inhibitor (Casin), restored Cdc42 activity to levels observed in young cells. This intervention produced long-lasting rejuvenation of HSC function in vivo, probably through epigenetic remodelling induced by modulation of Cdc42 activity. Subsequent studies extended these observations by showing that age-related alterations in protein organisation within HSCs are functionally relevant. Ageing is associated with changes in HSC polarity, with proteins becoming more symmetrically distributed within the cell. This loss of polarity is linked to altered differentiation potential and favours myeloid over lymphoid lineage commitment, thereby contributing to the age-associated myeloid bias of haematopoiesis. The Authors further demonstrated that systemic administration of CASIN to aged 75-week-old female C57BL/6 mice for four consecutive days significantly increased both mean and maximum lifespan. Treatment was also associated with reduced circulating levels of age-related inflammatory cytokines, including interleukin (IL)-1β, IL-1α and IFN-γ, and with rejuvenation of the epigenetic clock, as assessed by DNA methylation patterns in blood cells. Overall, these findings indicate that pharmacological reduction of Cdc42 activity in aged mice can extend lifespan and partially restore a more youthful haematopoietic and inflammatory profile. Importantly, the observation that transplantation of HSCs from CASIN-treated old mice reproduced lifespan-extending effects in untreated aged recipients further supports the concept that HSC function can influence organismal ageing beyond the haematopoietic compartment. These data suggest that age-related HSC dysfunction is not merely a marker of ageing of immune system but may actively contribute to systemic ageing processes [63,64].

From a longevity perspective, these findings suggest that the preservation of HSC fitness may contribute to immune resilience by maintaining a more balanced lymphoid–myeloid output, limiting inflammaging and sustaining protective immunity in late life. However, the available evidence remains largely experimental, and translation to human exceptional longevity requires caution. In humans, it remains to be clarified whether LLIs preserve HSC function more effectively, whether they resist age-related myeloid skewing, or whether they compensate for it through other mechanisms, such as better control of inflammation, preserved cytotoxic surveillance or favourable immunobiographical trajectories. Thus, HSCs should not be regarded as isolated “longevity cells”, but as part of a broader haematopoietic and immune architecture that may influence the balance between immune decline, chronic inflammation and healthy survival [61].

### 4.2. Clonal Haematopoiesis and Genomic Stability in Exceptional Longevity

Returning to myeloid bias, the picture is further complicated by clonal haematopoiesis of indeterminate potential (CHIP), an age-associated condition characterised by the expansion of haematopoietic stem cell clones carrying somatic mutations that confer a selective growth advantage. As many CHIP-associated mutations arise in HSCs and can favour the expansion of myeloid-biased clones, CHIP provides a further mechanistic link between ageing of the haematopoietic compartment, inflammatory remodelling and late-life disease. Genetically distinct blood cell populations generated through this process are associated with a more than tenfold increased risk of haematological malignancies, as well as with a higher risk of cardiovascular disease [65]. CHIP has also been investigated in exceptionally long-lived individuals. As previously stated, in Italian semi-supercentenarians and supercentenarians, Garagnani et al. [53] identified a distinctive genomic profile associated with more efficient DNA repair, as reflected by both germline variants and patterns of somatic mutations. These findings suggest that preserved genome maintenance and a reduced burden of potentially deleterious somatic mutations, including CHIP-associated alterations, may represent one component of healthy exceptional longevity. Thus, in the context of extreme survival, the ageing of HSCs should be interpreted not only in terms of lineage skewing and myeloid bias, but also in relation to clonal dynamics, somatic mutation burden and the capacity to maintain genomic stability across the life course.

### 4.3. Thymic Involution and Adaptive Immune Ageing

The thymus is relevant to longevity because it is the primary site of T cell development and is therefore central to the maintenance of adaptive immune competence across the life course. Although it was long regarded as a largely dispensable organ in adult life, this view has been progressively revised. The thymus generates naïve T cells, which are required to preserve T cell receptor diversity, sustain responses to newly encountered antigens, support cancer immunosurveillance and maintain immune flexibility. With ageing, the thymus undergoes marked involution: in humans, most thymic epithelial and lymphoid tissue is progressively replaced by adipose tissue after puberty, and thymic T cell output declines substantially, reaching only a small fraction of childhood levels in later life. This decline contributes to contraction of the naïve T cell compartment, reduced immune repertoire diversity, expansion of memory and terminally differentiated clones, and impaired responses to infections, vaccines and malignant transformation. In this sense, thymic involution may represent one of the structural drivers of immune ageing [66,67,68]. Studies in centenarians consistently indicate that advanced age is associated with a profound reduction in recent thymic output and a marked remodelling of the T cell compartment. Nasi et al. [69] have reported that T cell receptor rearrangement excision circle-positive cells were undetectable in most centenarians, together with extremely low numbers of naïve T cells. These findings suggest that, in extreme old age, the maintenance of T cell immunity depends much less on continuous thymic generation of new naïve cells and much more on peripheral T cell homeostasis, memory-cell persistence and antigen-driven immune remodelling.

### 4.4. Thymic Function, Emerging and Globalised Antigenic Challenges in Exceptional Longevity

The reasons underlying thymic involution are not fully understood, although an evolutionary explanation has often been proposed. According to this view, by the time puberty is reached, individuals have already been exposed to many of the microorganisms that characterise their local environment. Under these circumstances, maintaining a highly active thymus and continuously generating large numbers of new naïve T cells may become biologically inefficient, particularly if many of these cells are unlikely to encounter novel antigens. After puberty, encounters with entirely new pathogens may have been less frequent in ancestral environments, whereas long-lasting immune memory against previously encountered environmental microorganisms became increasingly important. From this perspective, the progressive decline in thymic activity could reflect a shift in immune investment: from the generation of new T cell specificities towards the maintenance and regulation of memory responses that provide protection against familiar pathogens [12].

However, this evolutionary assumption must be reconsidered in the modern world. In contemporary societies, microbial globalisation, international travel, urbanisation, ecological disruption, migration, zoonotic spillover and rapid pathogen spread have substantially increased the likelihood that individuals will encounter novel or previously uncommon infectious agents throughout life. Thus, the idea that exposure to new pathogens becomes rare after puberty is no longer fully applicable. In this context, progressive thymic involution may become maladaptive, because reduced thymic output limits the generation of new naïve T cells and narrows the capacity of the ageing immune system to respond effectively to emerging pathogens, new vaccines and changing antigenic landscapes. A paradigmatic example was provided by the severity of the COVID-19 pandemic, which showed how, in a world characterised by microbial globalisation, encounters with novel or rapidly spreading pathogens may occur at any stage of life [12,70].

The importance of the thymus is also supported by experimental and clinical observations. Classic neonatal thymectomy studies in mice showed that the absence of the thymus profoundly impairs lymphoid development and increases susceptibility to infection, establishing the idea that the organ is essential for T cell generation. More recent human data suggest that thymic integrity may remain clinically relevant even in adulthood. In fact, thymectomy has been associated with increased mortality and cancer risk, while smaller thymic size in people with intact but age-involuted thymus has been correlated with poorer survival and higher risk of cancer and cardiovascular disease. These findings do not prove causality, but they support the hypothesis that preserved thymic function may contribute to healthier ageing by maintaining immune surveillance and adaptive immune reserve [71,72,73,74,75].

From the perspective of exceptional longevity, the thymus should not be interpreted as a simple “longevity organ”, nor should thymic regeneration be presented as a proven anti-ageing intervention. Rather, its relevance lies in its potential contribution to immune resilience. Individuals with favourable ageing trajectories may benefit from a better-preserved balance between thymic output, peripheral T cell homeostasis, memory-cell maintenance and control of chronic antigenic stimulation. Conversely, excessive thymic involution may accelerate immune narrowing, reduce the capacity to respond to novel pathogens and vaccines, and favour a compensatory expansion of differentiated T cell populations shaped by lifelong antigenic exposures, including cytomegalovirus (CMV). Thus, thymic function is important for longevity not because it alone determines lifespan, but because it contributes to the adaptive capacity of the immune system, which is a key component of healthy ageing and resistance to late-life disease. However, its role in human exceptional longevity remains to be defined by mechanistic and longitudinal studies rather than inferred solely from thymic size or experimental models [71,73].

## 5. Immunosenescence

### 5.1. Immune Ageing, Inflammaging and Cellular Senescence

An effective immune system is essential for healthy ageing, as it protects the host against infectious agents, contributes to the control of age-associated cellular damage, and supports the recognition and elimination of both senescent and malignant cells. In this review, we use immunosenescence as an umbrella term encompassing both immune ageing, defined as the progressive remodelling and functional alteration of immune responses with age, and inflammaging, the chronic, low-grade, sterile inflammatory state that commonly accompanies ageing. These two processes are closely interconnected: immune ageing can promote inflammaging, while persistent inflammation may in turn accelerate immune dysfunction. Immune ageing is a complex, dynamic and heterogeneous process. Rather than representing a uniform decline in immune competence, it involves the deterioration of some immune functions, the preservation of others and, in some cases, the enhancement of specific immune responses, with substantial interindividual variability. Overall, these age-associated changes may contribute to the increased incidence and severity of emerging infections and to the reduced effectiveness of vaccination in older adults. Typical features include impaired responses to new antigens and vaccines, contraction of the naïve T and B cell compartments, and expansion of antigen-experienced memory and terminally differentiated cells. Persistent CMV infection is a major driver of the peripheral accumulation of effector-memory and T effector memory cells re-expressing CD45RA (TEMRA), although it does not appear to fully explain the age-related loss of naïve T cells [12,76].

A further relevant aspect is the interaction between immune competence and cellular senescence. Cellular senescence is a state of stable cell-cycle arrest induced by different forms of stress, including DNA damage, telomere shortening, oncogene activation, oxidative stress and chronic inflammation. Although senescence may initially serve as a tumour-suppressive and tissue-protective mechanism, senescent cells can accumulate with age when immune clearance becomes inefficient. These cells may acquire a senescence-associated secretory phenotype (SASP), characterised by the production of cytokines, chemokines, growth factors, proteases and extracellular matrix-remodelling mediators, thereby amplifying inflammation and tissue dysfunction. A competent immune system therefore contributes not only to host defence, but also to immune surveillance against senescent and malignant cells. Efficient removal of senescent cells may limit SASP-driven inflammation and tissue damage. Conversely, inflammaging contributes to frailty, neurocognitive decline and several age-related pathological conditions [12,77].

This view also implies a reciprocal relationship between immune ageing and systemic ageing. The ageing immune system should not be regarded only as a passive target of chronological ageing, but also as an active contributor to systemic functional decline. Jang et al. [78] recently emphasised that older individuals acquire biochemical and functional immune-cell alterations that may lead to chronic inflammation, impaired pathogen immunity and organ dysfunction. These dysfunctional immune states can promote multi-organ dysfunction and systemic ageing, whereas improving immune function may increase resilience and extend healthspan. This concept is particularly relevant to exceptional longevity, because it suggests that preserved immune competence and controlled inflammation are not merely consequences of healthy ageing, but may instead actively contribute to maintaining tissue homeostasis, reducing frailty and supporting long-term healthspan. The same review consistently highlights that age-associated immune dysfunction can disturb tissue architecture, increase fibrosis, promote myeloid-derived inflammation and impair interactions between immune and stromal cells. Thus, the immune system may represent both a marker and a driver of biological ageing. In centenarians and the oldest centenarians, successful immune ageing may therefore reflect not the absence of immunosenescence, but the capacity to limit its systemic consequences through preserved surveillance, regulated inflammation and resilient immune–tissue communication.

### 5.2. Determinants and Consequences of Immune Ageing

Chronological age is only one determinant of immune-system variability. Genetic background, sex, lifelong environmental exposures, and cumulative antigenic stimulation, including CMV infection (see next paragraph) and vaccination history, may also play major roles; together, these factors contribute to an individual’s immunobiography. Age-associated epigenetic remodelling of lymphocytes is, in fact, strongly influenced by this immunobiography. Although research on immune ageing has traditionally focused on T and B lymphocytes, innate immunity is also affected by ageing. Changes occur in the abundance, phenotype, tissue distribution, and functional capacity of neutrophils, monocytes, and dendritic cells for an extensive discussion, see [12,79,80].

An additional dimension of lifelong antigenic exposure is provided by the recently proposed concept of the silent infection load, which encompasses the cumulative immunological burden generated by clinically silent microbial encounters, including asymptomatic infections, intermittent reactivation of latent pathogens and other forms of low-grade persistent microbial activity. Although individually mild or clinically undetectable, such events may produce repeated episodes of immune activation and inflammatory stress over the life course. Their cumulative effects have been proposed to contribute to immune remodelling and inflammaging through mechanisms that include endothelial perturbation, mitochondrial stress, complement activation and progressive micro-damage. Persistent infections such as CMV and Epstein–Barr virus may represent important components of this lifelong infectious burden. From an evolutionary perspective, microbial persistence may also represent a trade-off, whereby sustained immune surveillance and host–microbe coexistence provide benefits such as immune readiness and cross-protective responses earlier in life, while progressively increasing inflammatory and energetic costs at older ages [81].

This hypothesis is particularly intriguing in the context of exceptional longevity. Centenarians, semi-supercentenarians and supercentenarians have experienced an exceptionally long period during which asymptomatic infections, latent pathogen reactivation and repeated antigenic exposures could accumulate. Their survival to extreme ages therefore suggests that exceptional longevity does not necessarily require avoidance of lifelong infectious pressure. Rather, it may depend, at least in part, on the capacity to contain or compensate for its potentially detrimental consequences while maintaining effective immune surveillance. In this perspective, the biological effect of cumulative infectious exposure may be determined not only by the magnitude of the antigenic burden itself, but also by the individual immunobiographical trajectory and by the ability to maintain a functional balance among immune memory, inflammatory responses, regulation and cytotoxic defence. Whether exceptional LLIs differ from less long-lived people in their capacity to tolerate or adapt to the silent infection load remains an intriguing hypothesis that requires direct investigation.

However, immunosenescence is associated with increased susceptibility to infections and weaker vaccine-induced protection, whereas preserved immune function may help delay the onset or progression of major chronic diseases, including cancer, cardiovascular disease and Alzheimer’s disease. Accordingly, healthy ageing and longevity are generally associated with better regulation of chronic inflammation and lower levels of inflammaging. Overall, immune competence represents a major determinant of both lifespan and healthspan [12,80,82].

### 5.3. Sex-Associated Trajectories of Immune Ageing and Inflammaging

As discussed in the first section, immune responses are generally stronger in females than in males, and this may contribute, at least in part, to the greater life expectancy and higher prevalence of exceptional longevity observed in females. Sex-specific trajectories of immunosenescence have been described, but whether these differences persist at the most advanced ages, including in centenarians and semi-supercentenarians, remains insufficiently explored. Interpretation is also limited by the marked imbalance between females and males in centenarian cohorts, a disproportion that becomes even more pronounced with increasing age [83,84]. Available data nevertheless suggest that immune ageing progresses differently in males and females. In a study of 356 healthy Japanese individuals aged 20–90 years, Hirokawa et al. [85] analysed lymphocyte subsets by three-colour flow cytometry and assessed T cell proliferation and cytokine production after anti-CD3 stimulation. Ageing was associated with reduced numbers of total T cells, CD8^+^ T cells, CD4^+^CD45RA^+^ T cells, CD8^+^CD28^+^ T cells and B cells, together with impaired T cell proliferative capacity. However, the decline in most of these parameters was more pronounced in males than in females. Conversely, CD4^+^ T cells, CD4^+^CD45RO^+^ T cells, NK cells and the CD4/CD8 ratio increased with age, with a steeper increase in females. Both the T cell proliferation index and a composite T cell immune score declined with age, again more markedly in males than in females, suggesting a faster deterioration of selected adaptive immune functions in males.

Consistent with these observations, Márquez et al. [86] combined ATAC-seq, RNA-seq and flow cytometry in peripheral blood mononuclear cells (PBMCs) from 172 healthy adults aged 22–93 years. The study identified a shared epigenomic signature of ageing, characterised by loss of naïve T cell features and increased monocyte and cytotoxic cell programmes. These changes were stronger in males and were accompanied by a male-specific decline in B cell-associated loci. Age-related epigenomic remodelling appeared to occur in two waves: an initial rise in late adulthood with broadly similar timing in both sexes, followed by a second wave that occurred earlier and with greater intensity in males. After the age of 65, sex differences became more pronounced, with males showing higher innate and pro-inflammatory activity and lower adaptive immune signatures.

Inflammatory markers also appear to follow sex-biased trajectories. Men over 65 years tend to show higher IL-6 levels and related inflammatory markers than age-matched females, a pattern that may contribute to lower male survival. Although individual cytokine values vary across age groups and studies, the overall pattern supports the view that males accumulate innate and inflammatory alterations earlier and more intensely than females, whereas premenopausal females may be relatively protected from inflammaging [87,88].

These sex-related differences are not determined by hormones and sex chromosomes alone. They are also shaped by the exposome, including pollutants, diet, occupational exposures, psychosocial stress and other life-course environmental factors. Such exposures may accelerate inflammaging in a gender-dependent manner: men may be more susceptible to inflammation driven by outdoor pollutants and occupational exposures, whereas women may be more affected by indoor pollutants and endocrine-disrupting chemicals [88,89].

Overall, the available evidence suggests that inflammaging in males is often characterised by earlier and stronger inflammatory and innate immune activation, while females tend to maintain more favourable immune trajectories for longer.

### 5.4. Gut Microbiota, Immune Ageing and Exceptional Longevity

The gut microbiota may also contribute to shaping immunosenescence, especially through its influence on the chronic pro-inflammatory state that characterises ageing. The human gut microbiota changes continuously across the life course. Its composition is influenced from early life by delivery mode, nutrition, environmental conditions, geographical setting, medication exposure and the ageing process itself. Some studies suggest that individuals who reach very advanced ages in relatively good physical and cognitive condition may harbour microbial communities enriched in taxa considered favourable for healthy ageing and immune response, including *Bacteroides*, *Lactobacillus, Akkermansia*, *Methanobrevibacter*, members of the *Ruminococcaceae* family, and other potentially beneficial organisms. However, the picture is not uniform, as some butyrate-producing genera, such as *Faecalibacterium*, *Roseburia* and *Eubacterium*, have been reported at lower levels in centenarians. Microorganisms with potentially beneficial functions may generate metabolites, especially short-chain fatty acids (SCFAs), which contribute to immune regulation, preservation of intestinal barrier integrity and reduction of oxidative and inflammatory stress. An increased abundance of SCFA-producing bacteria is generally associated with reduced intestinal permeability, partly because SCFAs provide an energy source for colonocytes and support epithelial barrier function. Through this mechanism, they may limit the translocation of inflammatory stimuli from the gut into the systemic circulation, thereby contributing to lower chronic inflammation [8,9,90,91,92].

Further insight comes from the multiomics study of the individual with the most extreme documented lifespan. In this case, the gut microbiota was analysed as one component of the biological dissociation between extreme chronological age and preserved health [93]. Her faecal microbiota was compared with that of 445 control individuals, including 250 women and 195 men aged 61–91 years who were not receiving antibiotics. The most notable finding was the unusually high microbial richness, reflected by α-diversity, which captures microbial diversity within an individual sample, suggesting a diverse gut ecosystem despite extreme age. β-diversity, which measures differences in microbial community composition between samples or individuals, also showed that her gut microbiota was clearly distinct from that of the control population.

Taxonomically, the most evident feature was the enrichment of *Actinobacteriota*, largely driven by increased *Bifidobacteriaceae* and particularly by the genus *Bifidobacterium*. This is noteworthy because *Bifidobacterium* typically decreases with ageing, whereas higher levels have been described in centenarians and oldest centenarians. The authors interpreted the abundance of *Bifidobacterium* as potentially favourable, given its association with anti-inflammatory activity and with the production of metabolites such as SCFAs and conjugated linoleic acid. This microbial profile was consistent with the low-inflammatory state and favourable lipid-metabolic pattern observed in this supercentenarian. Diet may have contributed to this configuration, as she reportedly consumed approximately three yoghurts per day containing bacteria that can promote bifidobacterial growth. Together with her Mediterranean dietary pattern, this suggests a possible long-term interaction between diet, microbiota and healthy ageing. Other features, including lower levels of potentially pro-inflammatory bacteria, were also compatible with a favourable ageing phenotype [92].

Overall, current evidence suggests that supercentenarians may show high microbial diversity and enrichment of bacterial taxa linked to anti-inflammatory and metabolically favourable functions. However, available data remain limited, often deriving from very small cohorts or single-case reports [92,93]. Therefore, no universal gut microbiota signature of supercentenarian longevity can yet be defined. Larger, internationally coordinated studies are required to determine whether these microbial profiles are causes, consequences or correlates of exceptional human longevity.

Evidence indicates that sex is an important source of variability in gut microbiota composition. Sex-related differences have also been reported in the bacteria-to-human cell ratio, which has been estimated to be approximately 1.3 in men and 2.2 in women. Accordingly, sex is increasingly considered not merely as a potential confounder, but as a biologically relevant variable that should be specifically addressed in gut microbiota research. A recent review discussed sex-related differences in gut microbiota largely in relation to the effects of sex hormones. However, microbiota composition may also be shaped by gender-related factors, including differences in education, lifestyle, dietary habits and behaviour [94,95].

**Figure 2:**
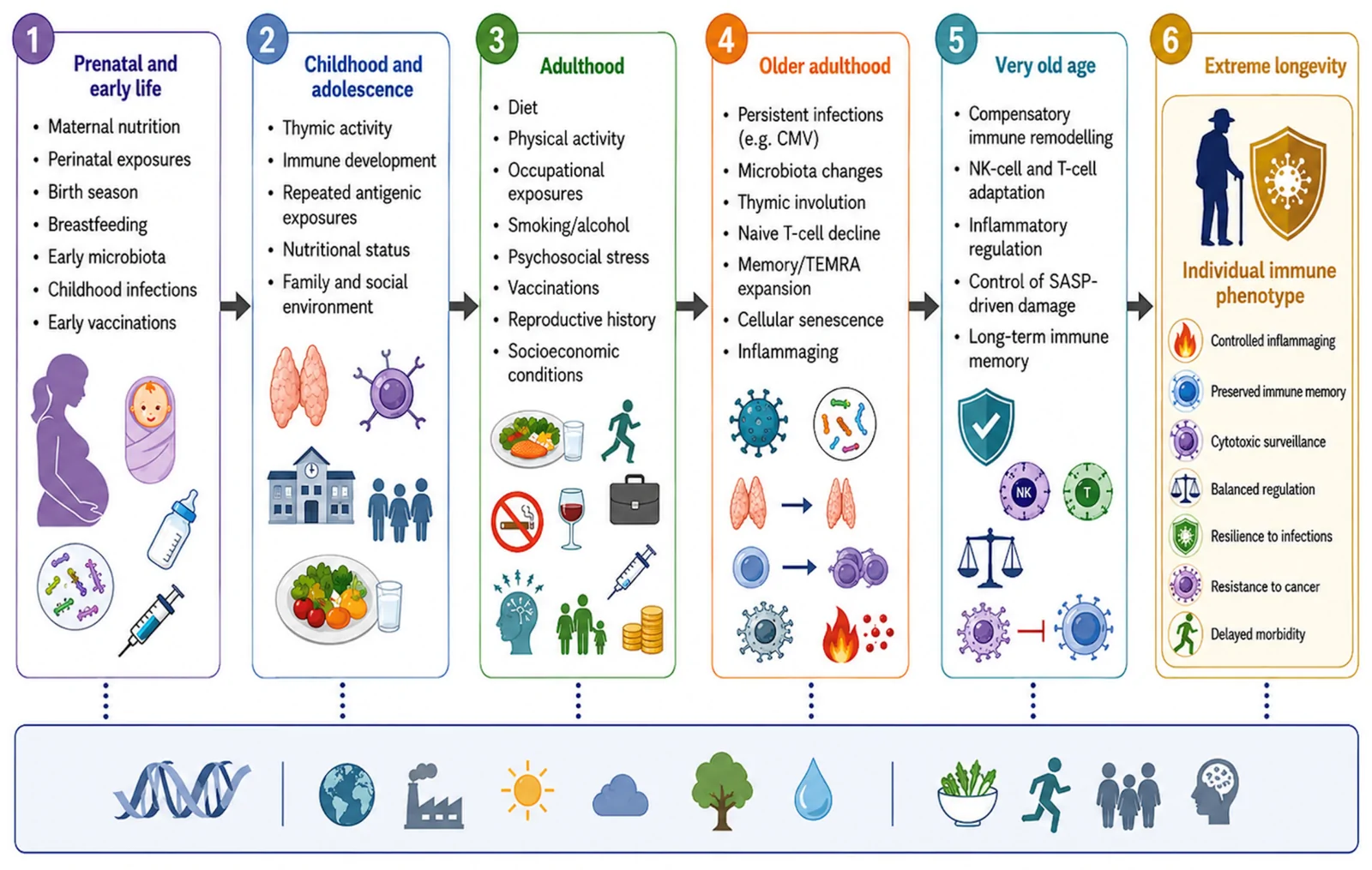
**Immunobiography of a centenarian.** The immune phenotype observed in centenarians and the oldest centenarians reflects the cumulative influence of genetic background, sex and lifelong exposures, including infections, vaccinations, microbiota, nutrition, lifestyle, environmental stressors and social context. Beginning in prenatal and early life, these factors shape immunological trajectories that are subsequently remodelled by antigenic stimulation, persistent infections such as CMV and other exposures throughout adulthood and old age. Their combined effects influence immune memory, inflammatory tone, thymic output, haematopoietic remodelling and cytotoxic surveillance. At extreme ages, the resulting phenotype therefore does not necessarily represent preservation of a youthful immune system, but rather a personalised adaptive equilibrium among immune attrition, memory, regulation, inflammation and cytotoxic defence. Immunobiography thus provides a framework for understanding the heterogeneous yet successful immune trajectories that may support exceptional longevity.

Figure 2 summarises how genetic background, exposome and cumulative immune experience may shape the immune phenotype observed in centenarians and in the oldest centenarians.

## 6. Immune Ageing in Extreme Longevity: NK Cells

### 6.1. Age-Associated NK-Cell Remodelling

Human peripheral blood NK cells are commonly classified according to CD56 and CD16 expression. The predominant CD56^dim^CD16^+^ subset, accounting for approximately 90% of circulating NK cells, consists of mature cytotoxic effectors with strong spontaneous killing activity and the capacity to produce IFN-γ after direct contact with susceptible target cells. In contrast, the less abundant CD56^bright^ subset expresses little or no CD16 and exerts mainly immunoregulatory functions through the release of cytokines and chemokines in response to IL-2, IL-12, IL-15, and IL-18. A third population, CD56^−^CD16^+^ NK cells, is characterised by limited proliferative and cytotoxic capacity, reduced expression of activating receptors, and a relatively inhibitory phenotype. Ageing is generally accompanied by an increase in the proportion, and in some studies also the absolute number, of circulating NK cells. This apparent numerical preservation, however, masks a substantial redistribution among subsets. CD56^bright^ cytokine-producing cells tend to decline, whereas the more differentiated CD56^dim^ population is maintained or expanded. CD56^−^CD16^+^ cells may also accumulate, particularly in the setting of chronic viral infections. Thus, the enlargement of the NK cell compartment in older adults should be interpreted as the result of the preferential survival and accumulation of mature, long-lived, and functionally specialised cells. Accordingly, NK cell ageing is better viewed as a process of selective remodelling than as a uniform functional deterioration. It involves the preservation of overall cell numbers, contraction of immature regulatory populations, enrichment of differentiated cytotoxic subsets, changes in receptor expression, and selective defects in intracellular signalling and cytokine responsiveness. These alterations are strongly influenced by chronic CMV infection and may contribute to the increased vulnerability of older individuals to infections, malignancy, inflammatory disease, and frailty [96].

### 6.2. NK-Cell Expansion in Centenarians and the Oldest Centenarians

In a Sicilian cohort, the percentage of circulating NK cells rose significantly with age, largely because of the expansion of the mature cytotoxic CD56^low^CD16^+^ subset. By contrast, the CD56^high^CD16^−^ cytokine-producing population showed only a non-significant downward trend. The associations were no longer significant after separate analysis of men and women, although NK cell percentages were generally slightly lower in women. Thus, ageing was associated with a shift towards a more cytotoxic NK cell profile. This pattern was also evident in centenarians, who retained high proportions of total NK cells, particularly the CD56^low^CD16^+^ subset, whereas CD56^high^CD16−cells tended to be less represented [97].

To define the immunophenotype of the oldest centenarians, Ligotti et al. [98] subsequently measured CD3^−^CD56^+^CD16^+^ NK cells in another Sicilian cohort (described in the next section). NK cell percentages increased markedly across the age range, and the association remained significant in both sexes after stratification, although it was stronger in men. Taken together with previous observations, these findings suggest that NK cell expansion is not necessarily detrimental in centenarians, including those at the extremes of human survival. Rather, it may reflect an adaptive reorganisation of the immune system that supports resistance to lifelong antigenic and environmental challenges.

More recently, Bertolazzi et al. [99] found that the association between age and NK cell increase was no longer statistically significant after adjustment for sex, CMV serostatus, and circulating IL-6 levels. This attenuation may reflect the close biological interrelationship among these variables rather than a simple statistical artefact. Indeed, advancing age was strongly associated with both CMV seropositivity and higher IL-6 concentrations, supporting their contribution to immune remodelling during ageing [100]. Despite the predominance of women among the oldest centenarians, NK cell counts were significantly lower in females. This difference may result from the combined influence of genetic and hormonal factors, together with a possible trade-off between cell number and function.

### 6.3. Sex-Associated Differences and CMV-Driven Remodelling of NK

Female NK cells may be fewer but more activated or functionally mature. One proposed mechanism involves the X-linked epigenetic regulator KDM6A, also known as UTX [101]. Because some immune-related genes escape complete X-chromosome inactivation, females may express higher levels of UTX in NK cells. This enhanced expression has been associated with stronger antiviral activity but lower cell numbers. Conversely, lower UTX expression in men has been linked to greater NK cell abundance but reduced IFN-γ production, suggesting that numerical expansion does not necessarily correspond to superior antiviral function.

Finally, ageing and persistent CMV infection exert partly distinct effects on the NK cell compartment [102]. However, this contribution could not be examined directly in the Sicilian study because CD57^+^ and NKG2C^+^ NK cells, the subsets most strongly associated with CMV-driven adaptive or memory-like differentiation, were not measured. Consequently, any selective expansion or phenotypic remodelling of these populations related to CMV could not be identified in our dataset.

## 7. Immune Ageing in Extreme Longevity: T Cells

### 7.1. Age-Associated T Cell Remodelling

Ageing is accompanied by a progressive decline in the number and function of circulating T lymphocytes, together with major changes in their phenotypic composition. As previously mentioned, the most consistent alterations are the reduction of naïve T cells, the accumulation of antigen-experienced memory and terminally differentiated populations, particularly within the CD8^+^ compartment, and the progressive restriction of T cell receptor diversity. These changes reduce the capacity to recognise novel antigens and contribute to the impaired responses to emerging infections and vaccination observed in older adults. Two major processes shape this remodelling: thymic involution (see Section 4) and lifelong antigenic stimulation. Persistent infections further influence the peripheral T cell compartment. CMV is particularly important because recurrent antigenic stimulation promotes extensive expansion of virus-specific clones, especially among CD8^+^ T cells. These expanded populations may occupy a considerable proportion of the available immunological space, limiting the maintenance of less differentiated cells. They commonly display an effector-memory or TEMRA phenotype, lose the co-stimulatory molecules CD27 and CD28, and acquire markers such as CD57 and the killer cell lectin-like receptor subfamily G member 1 (KLRG1). Although these cells generally have limited proliferative capacity and reduced functional plasticity, their high numbers may sustain a substantial overall antiviral response [103]. However, these T cells may acquire senescent or exhausted phenotypes that limit effective T cell responses. Although senescent and exhausted T cells share overlapping features and are both characterised by impaired proliferation after T cell receptor (TCR) activation, they differ in their molecular signalling pathways and secretory profiles.

Senescent T cells secrete abundant pro-inflammatory mediators, acquiring a SASP, and may therefore contribute, at least in part, to inflammageing. By contrast, exhausted T cells show reduced cytokine-producing capacity and marked expression of inhibitory immune receptors, such as PD-1 and CTLA-4, which further constrain their functional activity. In both conditions, CD8^+^ TEMRA cells are prominently represented. These cells lack expression of the co-stimulatory molecule CD28, which is required for full T cell activation. Senescent T cells also express CD57 and KLRG1, while retaining high cytotoxic potential through the production of perforin and granzyme (GZM). Because of this cytotoxic profile, they are thought to contribute to the pathogenesis of cardiovascular and other age-related diseases [12,104].

Coming back to contraction of TCR diversity, age-related loss of immune competence should not be attributed mostly to this contraction. Modelling and sequencing studies suggest that major gaps in the T cell repertoire are unlikely to emerge unless T cell numbers are severely depleted [12,105]. Other mechanisms are also important. Age-associated defects in lymphoid microenvironments, including lymph-node fibrosis, together with reduced IL-7 receptor signalling, may impair homeostatic maintenance and disturb clonal balance, particularly within the CD8^+^ compartment. Epigenetic changes at the IL-7 receptor (IL-7R) locus and altered transcriptional regulation may further reduce survival, self-renewal and long-term maintenance of aged T cells. These alterations should therefore be interpreted as the result of several interconnected processes rather than as simple cellular exhaustion. Persistent antigenic stimulation favours T cell exhaustion and terminal differentiation; telomere attrition limits replicative potential; and SASP-related inflammatory signalling reinforces inflammageing within the T cell compartment. Taken together, the findings described above place T cell dysfunction at the intersection of the broader triad of immune ageing: chronic antigenic pressure, replicative limitation and persistent inflammatory activation [12,106].

### 7.2. T Cell Adaptation in Centenarians

Centenarians represent a particularly informative model because they display profound T cell remodelling without necessarily developing overt immune failure. Their immune profile is not youthful, but neither does it simply represent a more severe form of immunosenescence. Instead, it combines marked depletion of some populations with selective preservation or expansion of others, suggesting that exceptional longevity is associated with adaptation rather than uniform immune deterioration. True naïve CD4^+^ and CD8^+^ T cells are extremely rare in most centenarians. Moreover, cells identified as naïve solely based on CD45RA expression may belong to antigen-experienced populations, as many also express memory-associated markers such as CD95. Accurate identification therefore requires multiparametric phenotyping using CD45RA or CD45RO together with CCR7, CD27, CD28, and CD95. The contraction of the naïve compartment is accompanied by reduced repertoire diversity and consequently a diminished ability to respond to previously unencountered antigens. Peripheral homeostatic mechanisms may partly compensate for the loss of thymic output. The IL-7/IL-7R system appears relatively preserved in centenarians, while increased IL-15 levels may support memory-cell survival and turnover. However, the expanded memory compartment may compete with residual naïve cells for these cytokines, potentially limiting their maintenance. Memory and terminally differentiated T cells accumulate prominently in centenarians, particularly among CD8^+^. Although these populations are frequently considered markers of immune deterioration, their significance at extreme ages is more complex. Some retain rapid effector responses and cytotoxic activity and may therefore contribute to antiviral and anti-cancer surveillance. Large clonal expansions do not necessarily indicate pathological transformation, as they generally remain under homeostatic control. CD4^+^ cells also undergo substantial changes. Naïve CD4^+^ cells decline, whereas memory cells predominate. Helper functions may be reduced because of altered activation pathways, lower IL-2 production, and less effective support for B cell responses. Nevertheless, centenarian lymphocytes may retain IL-2-binding capacity, and proliferative responses vary according to the stimulus, indicating that T cell function is not globally impaired. T regulatory cell (Treg) changes in centenarians remain less clearly defined. Their accumulation could help limit chronic inflammation and autoimmunity, but excessive suppression might weaken antimicrobial and anti-cancer responses. The balance between regulatory and effector populations may therefore be more important than their absolute numbers [107].

### 7.3. T Cell Heterogeneity and Adaptation in the Oldest Centenarians

Longevity appears to depend on the capacity to maintain an effective balance among immune attrition, memory, regulation, and cytotoxic defence. This balance may be even more pronounced at the extremes of human lifespan, when the immune profile of the oldest centenarians may offer valuable insight into how exceptional LLIs accommodate the cumulative effects of chronological ageing and persistent infections by CMV. Using flow cytometry, Ligotti et al. [108] examined the relative frequencies and absolute counts of immune cell populations, with particular emphasis on T cell subsets, together with circulating inflammatory markers, in 54 participants comprising 28 women and 26 men aged 19–110 years. Age- and CMV-associated features of immunosenescence varied considerably across individuals. The eight oldest centenarians had the smallest proportions of naïve T cells, consistent with their advanced age, and the greatest representation of TEMRA, largely in relation to CMV serological status. They also displayed increased concentrations of serum inflammatory markers, although the average levels were lower than those observed in the other participants aged over 90 years. Notably, some of the oldest centenarians retained percentages of naïve and TEMRA CD8^+^ T cells, as well as exhaustion- and inflammation-related markers, within ranges that are like those found in younger individuals. These observations highlight the pronounced heterogeneity of immune ageing, particularly at the extremes of lifespan. Such variability cannot be explained by chronological age or any single determinant, but probably reflects the combined influence of genetic background, persistent infections, environmental exposures, and lifelong immunobiography. Because everyone follows a distinct biological and experiential trajectory, immune ageing is also highly personalised. Moreover, when interpreted alongside current evidence, the coexistence of CMV seropositivity, expanded TEMRA populations, and increased inflammatory markers should not necessarily be regarded as detrimental in centenarians. In the context of exceptional longevity, these features may instead represent components of an adaptive immune configuration that remains compatible with survival to very advanced ages [108].

### 7.4. γδ T Cell Remodelling in Ageing and Exceptional Longevity

In addition to αβ T lymphocytes, γδ T cells have also been investigated in ageing and exceptional longevity, although far less extensively than their αβ counterparts. Across three studies, γδ T lymphocytes showed a distinctive pattern of age-related decline that was also evident in centenarians. Argentati et al. [109] reported that the absolute number of circulating γδ T cells was significantly reduced in older adults and centenarians, mainly because of a selective loss of Vδ2 cells, whereas Vδ1 cell numbers remained relatively stable. Consequently, the Vδ2/Vδ1 ratio became inverted in advanced age. Despite this numerical decline, γδ T cells from centenarians retained cytotoxic activity and preserved IFN-γ production, although they showed increased tumour necrosis factor (TNF)-α production and a reduced capacity for in vitro expansion, particularly within the Vδ2 subset. Colonna-Romano et al. [110] further demonstrated that γδ T cells from old individuals and centenarians displayed a state of enhanced basal activation, characterised by increased CD69 expression and spontaneous TNF-α production. Their response to isopentenyl pyrophosphate was partially impaired, suggesting that chronic exposure to an inflammatory environment may contribute to functional dysregulation. These abnormalities may compromise the ability of γδ T cells to participate effectively in the control and resolution of inflammatory responses. In a subsequent study, Colonna-Romano et al. [111] showed that γδ T cells from centenarians had a markedly reduced proliferative response to both selective and polyclonal stimuli. They were also more susceptible to apoptosis induced by Fas and TNF-α. Overall, these findings indicate that, although some effector functions remain preserved, γδ T cells in centenarians are numerically depleted, chronically activated, poorly proliferative, and prone to apoptosis, making them unlikely to compensate for the age-related decline of conventional adaptive immunity.

Lastly, Ligotti et al. [112] characterised age-associated changes in the two major γδ T cell populations, Vδ1 and Vδ2, together with their differentiation profiles defined by CD27 and CD45RA expression. The study included the sample previously described. All participants had previously undergone αβ T lymphocyte and NK cell immunophenotyping using blood obtained at the same recruitment visit. The proportions of naïve Vδ1 and Vδ2 cells declined with advancing age, with the association being particularly marked for the Vδ1 subset. By contrast, terminally differentiated effector-memory cells re-expressing CD45RA increased significantly within the Vδ1 compartment but not among Vδ2 cells. The highest Vδ1 TEMRA values were observed in the oldest centenarians, although considerable interindividual variability was present. Naïve and TEMRA Vδ1 cell frequencies were strongly correlated with the corresponding CD8^+^ αβ T cell populations identified in our previous analysis, whereas comparable associations were not found for CD4^+^ αβ or Vδ2 cells.

These findings suggest that the expansion of γδ TEMRA cells is not necessarily detrimental in centenarians, including those of the most advanced ages. Rather, it may reflect a selective remodelling of immunity. The parallel enrichment of Vδ1 TEMRA, CD8^+^ TEMRA, and NK cells may represent coordinated adaptive responses that help exceptionally long-lived individuals withstand cumulative lifelong challenges and attain extreme longevity.

## 8. Immune Ageing in Extreme Longevity: B Cells

### 8.1. Age-Associated B-Cell Remodelling

The number of circulating B cells declines with advancing age, particularly among centenarians, including the oldest individuals [100]. Ageing is also accompanied by a progressive remodelling of the B cell compartment, with a shift towards late/exhausted phenotypes and a relative reduction in naïve and switched memory B cells. Because switched memory B cells are central to the generation of effective protective humoral responses, this redistribution contributes to less efficient antibody-mediated immunity in older adults. Functionally, B cell ageing is characterised by a reduced capacity to undergo class-switch recombination and somatic hypermutation, resulting in the production of lower-affinity antibodies with diminished ability to bind antigens effectively [113,114].

In older individuals, immunoglobulin production tends to shift from naïve-associated isotypes, such as IgD and IgM, towards memory-associated isotypes, including IgG and IgA. This occurs alongside an impaired ability to generate high-affinity protective antibodies against newly encountered pathogens and vaccines. Moreover, ageing is associated with a reduction in naïve IgD^+^CD27^−^ B cells and an expansion of IgD^−^CD27^−^ double-negative (DN) late-memory B cells, a subset commonly regarded as having an SASP [113,115].

The reduction in switched memory B cells and the concomitant accumulation of late/exhausted B cell subsets have also been linked to the impaired expression of activation-induced cytidine deaminase (AID), the enzyme responsible for introducing DNA modifications at immunoglobulin loci. Reduced AID expression may result from lower levels of E2A, a transcription factor required for AID upregulation following CD40/CD154 interaction and exposure to T cell-derived cytokines. However, an intrinsic age-related impairment of CD40 signalling, independent of E2A, cannot be excluded. Studies analysing age-associated changes in the B cell repertoire at the single-clone level have yielded partly conflicting results, probably because of differences in the number of genes and sequences examined, as well as the relatively small size of the cohorts studied [103,114].

### 8.2. B-Cell Ageing in Centenarian Offspring and Oldest Centenarians

An interesting exception to the typical age-associated B cell remodelling has been described in centenarian offspring. Although these individuals show a reduction in total B cell counts, like that observed in older adults, they do not display the characteristic naïve-to-memory shift usually associated with ageing. They do not show the increase in IgD^−^CD27− DN late-memory B cells or the decrease in IgD^+^CD27^−^ naïve B cells typically found in older subjects; rather, their B cell profile resembles that of younger individuals. The absence of the age-associated expansion of DN B cells suggests that centenarian offspring may be relatively protected from exhaustion of the B cell compartment. Consistently, their serum IgM concentrations remain within the range observed in young individuals. Overall, these findings suggest preservation of a functional bone marrow B cell reservoir in centenarian offspring, despite the well-recognised decline in B cell generation with age [113,115].

With regard to total circulating B cell levels, Bertolazzi et al. [100] assessed B cell counts by flow cytometry in the same Sicilian cohort previously described. A multiple negative binomial regression model was used to evaluate the effects of age, sex, CMV serostatus and IL-6 levels on B cell counts. The study showed that the age-related decline in circulating B cells was not primarily driven by chronological age per se but was largely associated with CMV seropositivity and systemic inflammation, as reflected by IL-6 concentrations. When these variables were included as covariates in the multivariable model, age was no longer an independent predictor of B cell counts. This suggests that previously reported age-related effects on B cell numbers may, at least in part, reflect indirect age-associated processes rather than ageing itself. In this cohort, neither sex-related differences in B cell counts nor a shift towards B cell predominance were observed among the oldest centenarians. Taken together, these findings highlight the importance of considering chronic CMV infection and inflammaging when interpreting trajectories of immune ageing, particularly in extremely long-lived individuals [100].

### 8.3. Age-Associated B Cells in Extreme Longevity

A striking feature of the supercentenarian described by Santos-Pujol et al. [93] is the expansion of a B cell cluster with features of age-associated B cells (ABCs). This subset is known to accumulate with ageing [116]. In this woman, the ABC cluster shows high expression of recognised ABC-associated markers, including SRY-box transcription factor (SOX)5 and Fc receptor-like 2 (FCRL2, see below). Notably, MYC, a well-established oncogene, was the most highly overexpressed gene in these cells compared with naïve and memory B cells. This finding raises the possibility that her ABCs may be acquiring proliferative features and transitioning towards the recently described population of age-associated clonal B cells, which depend on MYC activity for proliferation and have been proposed, in mouse models, to precede B cell malignancies [93,117].

ABCs form a distinct subset of B lymphocytes, first described in 2011, that progressively increases with age [118]. Unlike conventional B cells, which are primarily activated through antigen recognition by the B cell receptor (BCR), ABCs are particularly responsive to innate immune signals, especially those mediated by TLRs such as TLR7 and TLR9. They have been implicated in chronic inflammation, immunosenescence, autoimmunity, altered responses to infection and, in selected settings, clonal B cell expansion [116]. Thus, they should not be regarded simply as “old B cells”, but rather as a discrete immune cell population with a characteristic transcriptional and functional profile. Their frequency is increased in several autoimmune conditions, including systemic lupus erythematosus: whereas ABCs account for approximately 1% of circulating B cells in healthy donors, their proportion may exceed 5% in patients with lupus [119,120].

ABCs are also considered a form of atypical memory B cell. They represent a class-switched, antigen-specific memory population with a transcriptomic profile that distinguishes them from other B cell subsets. Functionally, they may produce pro-inflammatory cytokines, including IL-12 and TNF-α, thereby contributing to inflammaging. A growing body of evidence also indicates that ABCs participate in antiviral immune responses during both acute and chronic infections. During acute viral infection, they may transiently increase in the circulation and, after resolution of the infection, persist long term in lymphoid tissues such as the spleen. Their effector functions include antibody and cytokine secretion, as well as the capacity to activate T cells. Overall, ABCs represent a relatively rare but persistent effector B cell subset with distinctive activation requirements, localisation patterns, transcriptional signatures, and functional properties [117,119,120]. For the routes of ABC generation see Castro et al. [116].

Phenotypically, ABCs are typically CD11b^+^ and CD11c^+^, with reduced or absent expression of CD21 and CD23. Expression of the transcription factor T-box expressed in T cells (T-bet) has been described as a defining feature of this subset. Within the T-bet-dependent ABC programme, SOX5 has been identified as one of the regulated genes. SOX5 is a transcription factor rather than a classical B cell surface marker; therefore, its expression reflects a specific transcriptional state rather than a conventional phenotypic identity. Increased SOX5 expression supports the classification of these cells as belonging to an age-associated or atypical B cell differentiation pathway, distinct from conventional naïve or memory B cell compartments. As transcription factors regulate broader gene-expression programmes, SOX5 upregulation suggests that these cells have acquired a specialised ageing-associated differentiation state. However, its precise functional role in human ABCs remains incompletely defined. Although SOX5 has been linked to altered B cell differentiation and has been reported as upregulated in some B cell lymphoma contexts, its expression should be interpreted primarily as an ABC-associated transcriptional marker and not, by itself, as evidence of malignant transformation [121,122].

### 8.4. Molecular Features and Potential Clonal Evolution of ABCs

FCRL2, also known as CD307b, is a transmembrane surface receptor expressed by selected B cell subsets, with preferential expression in human memory B cells. It belongs to the Fc receptor-like family, whose members have important immunoregulatory roles in B cell biology. In this setting, FCRL2 helps distinguish age-associated or atypical B cells from conventional naïve B cells. Its expression is generally associated with differentiated, antigen-experienced, or memory-like B cell states, supporting the interpretation that these cells have undergone previous immune stimulation. Functionally, FCRL2 can modulate BCR signalling. It contains inhibitory signalling motifs, including ITIM-like sequences, and experimental evidence indicates that, when co-engaged with the BCR, it can dampen BCR-mediated activation. Thus, FCRL2 likely acts as an immunomodulatory receptor that fine-tunes the activation threshold of memory-like B cell [123].

The marked overexpression of MYC in the ABC cluster is particularly noteworthy. MYC is a major regulator of cell growth, metabolism, protein synthesis, and proliferation. As a canonical oncogene, excessive or dysregulated MYC activity can contribute to malignant transformation, including within B-cell lineages. However, the central point is not that this supercentenarian necessarily has a B cell malignancy. Rather, the authors make a cautious biological inference: the expansion of ABCs, their expression of ABC-related markers, and the strong upregulation of MYC may indicate that these cells are acquiring proliferative properties. This observation suggests a possible transition towards an age-associated clonal B cell state, resembling MYC-dependent populations described in mouse models as potential precursors of B cell malignancy. Nevertheless, this remains a hypothesis and should not be interpreted as direct evidence of cancer [93,116,124].

In conclusion, ABCs have an ambivalent role in immunity. On the one hand, they may contribute to host protection in the context of infection. On the other, their pro-inflammatory activity, association with autoimmunity, and potential for clonal expansion indicate that, under certain conditions, they may also participate in pathological processes.

## 9. T and B Cell Transcriptome in Extreme Longevity

Using single-cell sequencing, Deng et al., [125] analysed 387,762 cells from the human thymus and peripheral blood of young and older individuals, providing an integrated view of thymic involution and peripheral T cell ageing. In the thymus, ageing was associated with reduced T-lineage potential in early thymic progenitors, together with a relative increase in innate lymphoid lineage potential. The aged thymus was also characterised by the enrichment of mature T cells showing reduced expression of SOX4, a transcription factor involved in early T cell development and thymocyte differentiation, and by more pronounced inflammatory transcriptional profiles. In parallel, thymic epithelial cells were depleted, with reduced expression of tissue-restricted antigens, suggesting impaired thymic support for central tolerance and naïve T cell generation. At the peripheral level, the study identified transcriptional signatures of T cell ageing and developed an immune-age prediction model based on naïve T cells. The authors also proposed CD38 as a marker of recent thymic emigrants, providing a potential tool to estimate residual thymic output. Single-cell T cell receptor sequencing further revealed age-associated changes in TCR repertoire organisation, particularly within memory and effector T cell compartments, together with expansion of virus-specific T cell clones. Overall, these findings indicate that thymic involution contributes directly to immune ageing by limiting the generation of naïve T cells, reducing TCR diversity, and favouring the accumulation of oligoclonal memory and terminally differentiated T cell populations, including TEMRA cells [125].

Hashimoto et al. [126] used single-cell RNA sequencing to characterise 61,202 PBMCs obtained from seven supercentenarians and five younger controls. Their analysis identified a pronounced enrichment of cytotoxic CD4^+^ T cells as a distinctive feature of extreme longevity. Single-cell T cell receptor sequencing in two supercentenarians further showed that these cells had undergone extensive clonal expansion, with the dominant clonotypes representing 15–35% of the total CD4^+^ compartment. Although the degree of cytotoxic activity varied across cells, their transcriptional profile closely resembled that of conventional CD8^+^ cytotoxic T lymphocytes, indicating that they had adopted a CD8-like effector programme while preserving CD4 expression. Functionally, these cells released IFN-γ and TNF-α following ex vivo stimulation. The authors therefore proposed that the expansion of cytotoxic CD4^+^ T cells may represent an immune adaptation that supports protection against infections and malignancies in exceptionally long-lived individuals. B cells were significantly under-represented in supercentenarians compared with controls. The median proportion of B cells among the seven supercentenarians was approximately 2%, markedly lower than the 11% observed in the control group. This reduction was further confirmed by fluorescence-activated cell sorting (FACS) analysis performed in four supercentenarians and three controls. B cells were then subdivided into three clusters, designated BC1, BC2 and BC3. BC1 was consistent with a naïve B cell phenotype, based on the expression of IGHD, an immunoglobulin isotype present before class-switch recombination, together with the lack of the activation marker CD27. BC2 showed features of resting memory B cells, including expression of CD27, IGHG1 and IGHA. BC3 represented a minor population, detected across all donors, and displayed a plasma cell-like profile, characterised by high immunoglobulin expression, particularly IGHA and IGHG, together with CD38 expression and loss of CD20. Among these B cell subsets in PBMCs, the proportion of naïve B cells was significantly reduced in supercentenarians compared with controls. Memory B cells also tended to be less frequent in supercentenarians, although this difference did not reach statistical significance [126].

More recently, Hashimoto et al. [127] extended these observations by combining scRNA-seq, surface-protein and TCR profiling in T cells from eight individuals aged 70–99 years, ten centenarians and ten supercentenarians. CD4^+^ CTLs increased progressively across the three age groups, accounting for median proportions of 4.0%, 9.6% and 17.6% of CD4^+^, respectively. Integration with large public single-cell datasets further suggested that both the frequency and inter-individual variability of these cells increase markedly around the age of 100 years. Phenotypically, CD4^+^CTL differentiation appeared to proceed through sequential loss of the co-stimulatory molecules CD27 and CD28, with a CD27^−^CD28^+^ population representing an intermediate helper-to-cytotoxic state. Importantly, the fully differentiated CD4^+^CTLs retained a cytotoxic transcriptional programme without prominent expression of exhaustion-associated markers. TCR analysis revealed extensive clonal expansion, with the largest clone accounting on average for 33.3% of the CD4^+^CTL population, although the dominant TCR sequences were private to individual subjects. Ex vivo stimulation further revealed functional heterogeneity in cytokine production within individual clones. Taken together, these findings strengthen the interpretation of CD4^+^CTL expansion in extreme longevity as an adaptive response to repeated or persistent antigenic stimulation rather than simply a manifestation of terminal T cell dysfunction [127].

Consistent with these observations, Zhong et al. [128], in a study spanning childhood to supercentenarian ages, reported an increase in both cytotoxic CD4^+^ T cells and GZMK^+^GZMB^−^ CD8^+^ T cells in supercentenarians compared with individuals aged 60–80 years. These CD8^+^ cells have an intermediate or less terminally differentiated effector state rather than fully developed cytotoxic activity. Taken together, these findings support the view that the acquisition of cytotoxic properties by CD4^+^ T cells may help preserve antiviral and anti-cancer surveillance at the extremes of human lifespan.

Wang et al. [129] extended this picture through an integrated analysis of 87,215 PBMCs from seven supercentenarians, three centenarians, and four older controls, combining single-cell transcriptomics with FACS. Oldest centenarians showed enrichment of CD8^+^ effector-memory T cells and more differentiated B cell populations, whereas naïve T cells and Treg were more abundant in older controls. B cells were classified into naïve, memory, terminally differentiated B cells, and plasma cells. Compared with older controls, the longevity group showed enrichment of memory B cells and plasma cells, whereas naïve B cells were more abundant in the control group. Pseudotime analysis, a computational approach used to infer cellular differentiation trajectories from single-cell transcriptomic profiles, placed naïve B cells at early stages and plasma cells at later stages of B cell differentiation, suggesting a shift towards a more differentiated humoral compartment in centenarians and supercentenarians. At the transcriptional level, B cells associated with exceptional longevity showed a profile consistent with maintained humoral function and enhanced immune and metabolic activity. Taken together, these findings suggest that exceptional longevity is associated with adaptive B cell remodelling towards more differentiated states rather than simple B cell exhaustion [129].

Karagiannis et al. [130], investigated immune ageing and extreme longevity using newly generated single-cell profiles from seven centenarians, with a mean age of 106 years, together with publicly available datasets including seven additional centenarians and 52 individuals aged 20–89 years. Their analysis confirmed established age-related shifts from lymphoid to myeloid cells and from non-cytotoxic to cytotoxic populations. The lymphocyte compartment of the oldest centenarians showed an almost 50% reduction in non-cytotoxic lymphocytes, together with a relative enrichment of B cells. The authors also reported a marked shift from naïve to memory phenotypes within CD4 T cells and, to a lesser extent, within the B cell compartment. Transcriptional signatures associated with exceptional longevity included both age-related changes, such as increased expression of STK17A, a gene involved in the DNA-damage response, and gene-expression patterns uniquely detected in centenarian cells. Interestingly, among the immune compartments analysed, the oldest centenarians showed a less skewed distribution of cell types than younger individuals. This more balanced cellular composition may contribute to immune resilience, possibly reflecting a dynamic equilibrium in which immunocompetence is maintained through greater immune-cell diversity. Taken together with the enrichment of memory and cytotoxic populations, these findings suggest that centenarians had not escaped repeated antigenic exposures; rather, they may have retained the capacity to adapt to such challenges while preserving effective immune function [130].

Similarly, single-cell RNA-sequencing studies of Chinese centenarians and their offspring identified both numerical expansion and functional strengthening of cytotoxic T cell populations, particularly CD8^+^ T cells expressing CX3CL1 and GZMB [131].

Overall, these single-cell studies indicate that exceptional longevity is not characterised by uniform preservation of a youthful lymphocyte compartment, but by selective and coordinated T- and B cell remodelling. The T cell compartment appears biased towards cytotoxic and effector-memory programmes, whereas the B cell compartment shows evidence of differentiated humoral immune states, including memory B cells, plasma cells and terminally differentiated B cells.

The main single-cell transcriptomic studies addressing T- and B cell remodelling in centenarians and oldest centenarians are summarised in Table 2.

## 10. Immunological Memory in Extreme Longevity

### 10.1. Long-Term Persistence of Humoral and T Cell Memory

As discussed in Section 14.3, a sample of Sicilian centenarians, including semi- and supercentenarians, displayed high neutralising antibody titres against the 1918 H1N1 pseudotype virus. It is interesting to note that the titres were approximately 50-fold higher than those observed in 70-year-old controls. This finding suggests that immunological memory may, in selected circumstances, persist for more than a century [132].

On the other hand, extensive human studies have provided important evidence that both B and T cell memory can be maintained for remarkably long periods, even in the absence of repeated antigenic stimulation. The nature of the pathogen and the route of exposure are key determinants of the quality, localisation and durability of immune protection. Different classes of pathogens activate distinct innate immune sensors, antigen-presenting cells, effector pathways and inflammatory milieus, thereby shaping the differentiation and persistence of memory lymphocytes. Intracellular pathogens, for example, generally promote Th1- and cytotoxic T-cell dominated memory, whereas extracellular pathogens more often favour humoral immunity and Th17 type responses. The route of pathogen entry is equally important, as it determines the anatomical site of immune priming and favours the generation of compartmentalised memory populations. Respiratory viruses can induce lung- and airway-resident memory cells, which provide rapid local protection, whereas enteric infections tend to generate memory cells with gut-homing properties. Similarly, the route of vaccine administration can influence both the magnitude of the immune response and the tissue distribution of the resulting immune memory. Classical epidemiological observations indeed support the existence of exceptionally long-lived protective immunity. For instance, antibodies induced by inactivated poliovirus vaccination have been shown to persist for several decades, while smallpox-specific immune responses, including both antibodies and T cell responses, may still be detectable in older individuals many years after vaccination. Vaccinia-specific CD4^+^ and CD8^+^ T cells capable of producing IFN-γ and TNF have likewise been identified up to seven decades after immunisation. Although CD8^+^ T cell memory appears to decline gradually over time, CD4^+^ T cell memory seems to be considerably more stable. Overall, the generation, maintenance and longevity of T cell memory are controlled by complex molecular and metabolic programmes that distinguish short-lived from long-lived memory subsets. Although these populations share some core features, their persistence is regulated by distinct transcriptional, metabolic and epigenetic mechanisms [133,134,135,136,137,138,139,140,141,142].

### 10.2. Single-Cell Analysis of Vaccine-Induced T Cell Memory Across the Lifespan

High-dimensional single-cell technologies have recently been used to investigate CD4^+^ and CD8^+^ T cell memory induced by two temporally distinct vaccines: remote childhood vaccination with vaccinia/smallpox and recent vaccination against SARS-CoV-2. The study included individuals across the human lifespan, from adults to centenarians, including semi- and supercentenarians. Briefly, the investigators combined several complementary methods. Multiparametric flow cytometry was used to identify and quantify antigen-specific T cell populations and define their memory phenotypes. VASA-seq, a single-cell total RNA-sequencing approach, allowed detailed transcriptional profiling together with reconstruction of TCR repertoires, thereby linking gene expression patterns to clonal architecture. In parallel, single-cell metabolic regulome profiling was applied to characterise the metabolic programmes associated with distinct antigen-specific T cell subsets [143].

These approaches showed that antigen-specific T cell frequencies were also remarkably well preserved with age in the oldest centenarians. However, the quality of the response differed markedly according to antigenic history. Vaccinia-specific T cells showed stem-like and central memory features, preferential clonal expansion within long-lived memory compartments, and transcriptional and metabolic programmes enriched for fatty acid oxidation and oxidative phosphorylation. By contrast, SARS-CoV-2-specific T cells were mainly represented by effector and transitional memory subsets, with Th1–Th17 and Tc1–Tc17 polarisation, glycolytic metabolism, and inflammatory transcriptional signatures. Importantly, expanded and public TCR clonotypes persisted across age groups, while centenarians retained strong cytotoxic potential and high-affinity antigen recognition. Overall, these findings suggest that the durability and quality of human T cell memory are shaped more by antigen-imprinted clonal, transcriptional and metabolic programmes than by chronological age alone, offering a mechanistic framework for understanding vaccine-induced immunity across the lifespan [143].

## 11. Inflammaging

### 11.1. Mechanisms and Drivers of Inflammaging

In most individuals, inflammaging increases progressively with age and is reflected by increased circulating concentrations of biomarkers such as C-reactive protein (CRP), IL-6, TNF, CXCL9, and components of the NOD-like receptor family pyrin domain-containing protein 3 (NLRP3) inflammasome. This state of chronic low-grade inflammation is closely associated with multimorbidity, frailty, cardiovascular disease, and cancer. It is generally considered a consequence of the lifelong accumulation of multiple biological insults, including cellular senescence, mitochondrial impairment, genomic instability, gut microbiota alterations, and the progressive build-up of damage-associated molecular patterns. Cellular waste products, including misfolded proteins, dysfunctional organelles, and immunostimulatory nucleic acids, provide persistent endogenous danger signals that activate innate immunity. This phenomenon, often termed “garb-ageing,” perpetuates sterile inflammation through persistent stimulation of pattern-recognition receptors, including TLRs and inflammasomes, thereby maintaining chronic inflammatory activity even in the absence of infection [88,144]. CHIP has emerged as another age-related contributor to inflammaging. Current evidence indicates that mutant myeloid clones promote pro-inflammatory cytokine production and amplify pattern-recognition receptor signalling, although the precise causal mechanisms operating in humans have yet to be fully established [145].

### 11.2. Inflammaging and Inflammatory Resilience in Exceptional Longevity

An apparent paradox is observed in centenarians and, particularly, in the oldest centenarians. Although centenarians frequently exhibit circulating levels of inflammatory mediators that exceed those found in younger adults, they remain relatively protected from many inflammation-associated disorders, including cardiovascular disease and cancer. One explanation is that the biological consequences of elevated inflammatory signals are effectively buffered by robust compensatory mechanisms. These include enhanced regulatory immune networks, anti-inflammatory cytokines, soluble cytokine receptors, Tregs, and epigenetic and post-transcriptional mechanisms that collectively restrain excessive inflammatory responses. Thus, these studies support the concept that successful ageing is characterised not by the absence of inflammation but by the preservation of an efficient regulatory balance capable of limiting its harmful consequences. In agreement with this view, acute physical exercise has been shown to reduce circulating concentrations of some pro-inflammatory cytokines, particularly TNF, in centenarians compared with a resting condition [146,147,148].

In a Sicilian report, both the INFLA-score and the Systemic Inflammation Response Index (SIRI) showed a progressive increase with advancing age, indicating a gradual rise in systemic inflammatory burden [149]. The INFLA-score is a composite index that integrates circulating inflammatory and haematological parameters, including CRP, leukocyte count, platelet count and the neutrophil-to-lymphocyte ratio. SIRI, calculated as neutrophils × monocytes/lymphocytes, reflects the balance between innate inflammatory activation and lymphocyte-mediated immune regulation. An important exception was observed in semi-supercentenarians and supercentenarians, whose inflammatory values remained comparable to those observed in younger age groups, although with a great heterogeneity. These findings are consistent with previous reports describing the age-associated increase in chronic low-grade inflammation and further support the hypothesis that effective control of inflammatory responses contributes to healthy ageing and to exceptional longevity.

### 11.3. Sex and Life-Course Determinants of Inflammatory Trajectories

Sex-related differences also emerged. Females exhibited a less pronounced age-related increase in inflammatory indices than males. Specifically, the association between age and the INFLA-score was not statistically significant in females, whereas SIRI increased with age but to a lesser extent than in males. These observations are consistent with the well-established sexual dimorphism of immunosenescence whereby females generally maintain lower basal inflammatory activity, while older males display greater monocyte activation and a more pronounced pro-inflammatory profile. Such differences may contribute to the survival advantage of women observed throughout the lifespan. Several, not mutually exclusive, mechanisms may account for the relatively low inflammatory burden observed in semi- and supercentenarians compared with younger LLIs. These include lifelong adherence to healthy behaviours, such as a Mediterranean dietary pattern and regular physical activity, together with a favourable genetic background enriched for anti-inflammatory variants. Environmental factors, such as residence in small communities with lower exposure to pollution, together with strong family and social support capable of reducing chronic psychosocial stress, may also contribute [149]. Furthermore, more efficient clearance or tighter regulation of senescent cells, thereby limiting the production of SASP, could represent another important mechanism underlying the preserved inflammatory profile of these exceptional individuals [98].

### 11.4. Molecular and Functional Mechanisms of Inflammatory Resilience

The ability to regulate, rather than simply suppress, inflammation is likely to arise from multiple complementary mechanisms acting at the genetic, epigenetic and cellular levels. Human ageing is accompanied by changes in circulating microRNAs (miRNAs), which may reflect biological rather than chronological age and could serve as biomarkers of susceptibility to age-related inflammatory conditions. Senescent endothelial cells (ECs) have been proposed as one potential source of circulating miRNAs, linking vascular ageing, inflammation and systemic ageing trajectories. Accardi et al. [150] analysed a panel of four circulating miRNAs, miR-146a-5p, miR-126-3p, miR-21-5p and miR-181a-5p, selected for their involvement in pathways related to inflammation, endothelial dysfunction and cellular senescence, and for their potential association with a healthy ageing phenotype. Circulating levels of these miRNAs were measured in 78 healthy individuals ranging in age from 22 to 111 years. In parallel, extracellular levels of the same miRNAs were assessed in an in vitro model of senescent human ECs, to explore whether endothelial senescence could contribute to the circulating miRNA profile observed during ageing. Both the ex vivo and in vitro analyses showed a progressive age-related increase in the four miRNAs. Notably, however, the oldest centenarians displayed levels comparable to those observed in young individuals, although with a great heterogeneity. Overall, these findings support the relevance of selected circulating miRNAs as potential positive biomarkers of ageing trajectories [150]. Extracellular vesicles may represent an additional layer of immune regulation in centenarians, as their cargo of miRNAs, cytokines and proteins can modulate cell activation and intercellular communication, although their mechanistic role in exceptional longevity remains to be clarified [82].

Preservation of low NLRP3 inflammasome activation may represent another mechanism underlying the remarkable resilience of centenarians. In the general population, NLRP3 expression in lymphocytes increases substantially after approximately 65 years of age, consistent with the progressive establishment of inflammaging. In contrast, healthy centenarians retain NLRP3 expression levels like those observed in young adults, whereas unhealthy centenarians exhibit significantly higher expression. These findings suggest that the ability to prevent excessive activation of the NLRP3 inflammasome may contribute to protection against age-related diseases and support successful ageing [151,152].

A further functional dimension is provided by Sizzano et al., [153] who analysed lymphocyte responses to induced oxidative stress in seven semi-supercentenarians aged 105–107 years, their offspring, old controls and young individuals. Semi-supercentenarians showed a lower reactive oxygen species increase after oxidative challenge than old controls and offspring in most T, B and NK cell subsets, with values often comparable to young subjects. These findings suggest that the ability to buffer dynamic redox stress may represent a functional component of anti-inflammaging in extreme longevity, although confirmation in larger cohorts is required.

Because inflammatory trajectories are context-dependent and strongly influenced by the local exposome, resistance to inflammaging in centenarians, including the oldest centenarians, is unlikely to result from a single biological pathway. Instead, it appears to reflect distinct trajectories, some characterised by enhanced resilience through the ability to tolerate and counter-regulate inflammation, and others by mechanisms that prevent or limit its development.

**Figure 3:**
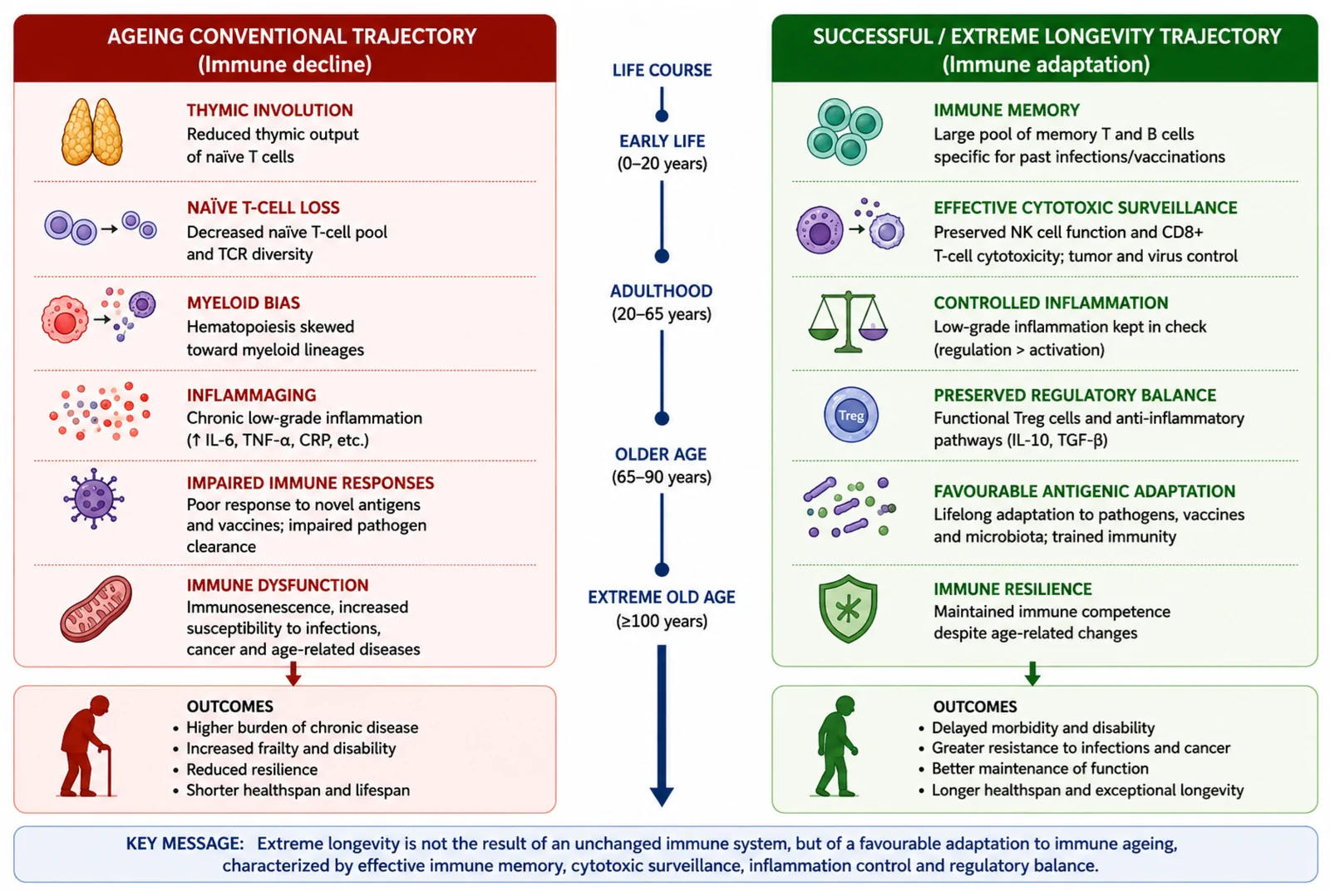
**Immune ageing versus immune adaptation in centenarians and, particularly, the oldest centenarians.** This schematic contrasts two possible immune trajectories in late life. The conventional ageing trajectory is characterised by progressive thymic involution, contraction of the naïve T cell compartment, haematopoietic myeloid bias and increasing inflammageing. These changes contribute to reduced immune flexibility, impaired responses to novel antigens and a higher burden of chronic inflammation. By contrast, the successful or extreme-longevity trajectory does not imply preservation of a youthful immune system, but rather a more favourable adaptation to immune ageing. In centenarians and in the oldest centenarians, immune competence may be supported by maintained immune memory, effective cytotoxic surveillance, controlled inflammatory activity and preserved regulatory balance. This adaptive equilibrium may contribute to resilience against infections, cancer and other age-related diseases, thereby supporting delayed morbidity and exceptional survival.

Figure 3 shows immune ageing versus immune adaptation in centenarians and, specially, the oldest centenarians. Indeed, as summarised in Table 3, immune features traditionally interpreted as detrimental markers of immunosenescence may acquire a different meaning in exceptional longevity, depending on their intensity, regulation and immunobiographical context.

## 12. From Morbidity Compression to Immune-Inflammatory Resilience in Extreme Longevity

### 12.1. Compression of Morbidity and Delayed Disease in Exceptional Longevity

Individuals who reach 100 years of age can postpone the onset of diseases associated with high mortality until the final phase of life. Nevertheless, a considerable proportion of centenarians, particularly females, may live for many years with chronic age-related conditions. Despite this burden, most centenarians appear to maintain functional capacity relatively well, with disability often becoming evident only in very advanced age. Individuals who attain even more exceptional ages, such as 107 years and beyond, seem to represent a more extreme expression of the compression of morbidity model. In these subjects, both disease burden and disability tend to be concentrated very late in life [36].

Andersen et al., [20] examined the relationship between survival age, morbidity and disability in centenarians aged 100–104 years, semi-supercentenarians aged 105–109 years, and supercentenarians aged 110–119 years. The cohort included 104 supercentenarians, 430 semi-supercentenarians, 884 centenarians, 343 nonagenarians and 436 controls, who were followed prospectively for a mean of 3 years, with a range from 0 to 13 years. The authors assessed the age at onset of individual diseases, overall morbidity, and cognitive and physical disability using survival analysis, across an age range marked by very large differences in survival probability among the oldest old. The results showed a clear gradient across age groups. Compared with nonagenarians and younger centenarians, the oldest centenarians generally developed major age-related conditions, including cancer, cardiovascular disease, dementia and stroke, at later ages. Cognitive and functional decline were also postponed in the longest-lived groups. Consistently, the hazard ratios for individual diseases progressively decreased with increasing survival age. The exceptional nature of these disease-free survival patterns is underscored by the fact that the age at onset of the conditions examined was substantially higher than the median age at death of the 1900 birth cohort. Across centenarian age groups, men showed better cognitive and physical function than women, in line with previous observations. Some diseases were almost absent among supercentenarians, including Parkinson’s disease and diabetes. This may indicate that such conditions are generally incompatible with survival to 110 years or beyond. However, because a healthy volunteer effect may have influenced all age groups, the possibility remains that supercentenarians with these diseases exist but were not represented in this cohort. The declining hazards for these conditions with advancing age also suggest that the most extreme survivors may be particularly informative for identifying factors associated with resistance to age-related diseases. When overall morbidity was considered, defined as the presence of one or more age-related diseases, the hazard of becoming morbid also declined progressively with increasing age among the centenarian groups. At the same time, the proportion of life spent with disease decreased as survival age increased. The median percentage of life lived with disease was 17.9% in controls, 9.4% in nonagenarians, 9.0% in centenarians aged 100–104 years, 8.9% in semi-supercentenarians and, according to regression analysis, 5.2% in supercentenarians. These findings are consistent with Fries’ compression of morbidity hypothesis [154], according to which the proportion of life spent with disease diminishes as individuals approach the limits of the human lifespan. The data are also compatible with Fries’ view that the exhaustion of organ reserve, understood as the progressive loss of the capacity to restore homeostasis, becomes a central determinant of maximum lifespan. With increasing age, a larger proportion of individuals escaped disease until the final three months or less of life: 3% of centenarians, 4% of semi-supercentenarians and 10% of supercentenarians. These percentages would probably be higher if a longer interval before death were used to define the period of morbidity compression. Overall, the older the age group, the shorter the period lived with disease. Taken together, these observations support the hypothesis that, beyond 100 years of age, survival is increasingly associated with relative resistance to age-related diseases and disability. In those reaching supercentenarian ages, death may be determined less by prolonged chronic disease than by the progressive exhaustion of functional and organ reserve, which limits the ability to withstand acute terminal events [20].

### 12.2. Biological Predictors of Successful Ageing: Telomere Maintenance and Inflammaging

In the longitudinal study by Arai et al., [21] three Japanese cohorts were combined to investigate biological predictors of successful ageing at extreme old age. The analytical sample included 1554 individuals: 536 very old community-dwelling subjects aged 85–99 years, 275 centenarians aged 100–104 years, 387 semi-supercentenarians aged 105–109 years, 22 supercentenarians aged 110 years or older, and 167 centenarian offspring with 167 unrelated family controls/spouses. The centenarian and extreme-longevity groups were predominantly female, with women representing 216/275 centenarians, 339/387 semi-supercentenarians and 20/22 supercentenarians. The study showed that telomere length behaved differently across age groups. In unrelated individuals, telomeres shortened with age up to 100 years, but after 100 years the trend appeared to reverse, with longer-than-expected telomeres in centenarians and particularly in the oldest centenarians. Centenarian offspring also maintained telomeres corresponding approximately to those expected in 60-year-old individuals from the general population. These findings suggest that preserved telomere maintenance may be a prerequisite or favourable background for exceptional survival. However, telomere length did not predict successful ageing once extreme longevity had already been achieved. Thus, telomeres may help individuals reach very old age, but they were not the main determinant of survival, functional capacity or cognition among centenarians and semi-supercentenarians. By contrast, inflammaging emerged as the most relevant biological domain. The inflammation score, based on CMV titre, IL-6, TNF-α and CRP, increased with age and predicted all-cause mortality both in the very old and in centenarians/semi-supercentenarians. In Cox models, the hazard ratio for mortality associated with inflammation was 1.89 in the very old and 1.36 in centenarians and the oldest centenarians. Inflammation was also the strongest predictor of capability and cognition among semi-supercentenarians, explaining more variance than chronological age or gender. Importantly, centenarian offspring had a lower inflammation index than their controls, suggesting that reduced inflammatory burden may characterise individuals predisposed to longevity before they reach extreme ages. The authors therefore propose that successful longevity is not explained by the absence of inflammation in centenarians, who may show elevated inflammatory markers, but rather by the delayed appearance or better control of systemic inflammation (Section 11.4.). In their interpretation, future centenarians may be protected for much of life by relatively low inflammatory activity; when inflammation rises late in life, it again becomes a strong predictor of mortality, disability and cognitive decline. This supports the view that inflammaging remains biologically relevant up to the limits of human lifespan. Thus, Arai et al. [21] suggest that long telomeres may contribute to the probability of reaching exceptional ages, but once this threshold has been crossed, the ability to maintain low or well-regulated inflammatory activity appears more closely linked to survival, preserved physical function and cognition.

### 12.3. Cognitive Resilience and Dementia in the Oldest-Old: Potential Links with Inflammation

Initiated in 2003, the 90+ Study is one of the largest longitudinal investigations of ageing and dementia in individuals aged 90 years and older, with more than 1600 participants enrolled. Age is the strongest risk factor for cognitive decline and major neurodegenerative disorders, including Alzheimer’s disease and dementia with Lewy bodies. Nevertheless, a subset of individuals maintains remarkably preserved cognition into extreme old age, making the oldest-old a particularly informative model in which to investigate mechanisms of cognitive resilience [155].

Findings from the 90+ Study have highlighted the marked heterogeneity of cognitive ageing after 90 years. Dementia affects a substantial proportion of individuals in this age group, while disability is even more common, with both conditions reported more frequently in women than in men. Importantly, clinicopathological correlations are often imperfect. Some individuals with dementia do not show a neuropathological burden sufficient to fully explain their cognitive impairment, whereas others remain cognitively preserved despite substantial Alzheimer-type, vascular or other age-related brain pathology. Many of these pathological processes are accompanied by neuroinflammatory responses, including microglial activation and cytokine signalling, which may influence their clinical expression. This dissociation indicates that dementia in the oldest-old depends not only on the burden of structural neuropathology, but also on biological mechanisms of resistance and resilience that may modulate its functional consequences [156].

More recent work from the 90+ Study has focused on the neuropathological correlates of preserved and superior cognition. In one analysis, autopsy findings from 102 cognitively unimpaired individuals who died at a mean age of 97.6 years were related to cognitive performance assessed during the final months of life. Cognitive testing had been performed approximately 2–12 months before death, at a mean age of 97.1 years. The aim was to identify brain characteristics associated not simply with the absence of dementia, but with preservation of high-level cognitive function into extreme old age. These individuals provide a particularly informative model of cognitive resilience, because they show that advanced chronological age and accumulation of neuropathological lesions do not inevitably translate into equivalent degrees of cognitive impairment [157].

From the perspective of exceptional longevity, an important question is whether the systemic immune-inflammatory adaptations associated with survival to very advanced ages may also contribute to cognitive resilience. The 90+ Study does not directly establish such an immune mechanism, but the dissociation between neuropathological burden and cognitive status provides a strong rationale for exploring this possibility. Chronic systemic inflammation may influence the ageing brain through several interconnected pathways, including activation of innate immune responses, microglial dysregulation and alterations in blood–brain barrier integrity. Conversely, more effective control of inflammaging and preservation of immune homeostasis could potentially attenuate the functional consequences of accumulating neuropathology. In this framework, cognitive resilience in the oldest-old may reflect not only intrinsic neuronal or synaptic reserve, but also the capacity to limit maladaptive neuroimmune and systemic inflammatory responses. Thus, the immune-inflammatory phenotype associated with exceptional longevity may be relevant not only to survival, but also to the preservation of brain function and resistance to the clinical expression of dementia. However, this relationship remains largely inferential, and direct longitudinal studies integrating peripheral immune profiles, neuroinflammatory markers, neuropathology and cognitive trajectories in the oldest-old are still needed.

### 12.4. Immune Resilience as a Framework for Healthy Ageing and Exceptional Longevity

A complementary framework has recently been proposed through the concept of immune resilience, defined as the capacity to preserve or rapidly restore immune functions that promote disease resistance while controlling inflammation during infectious and non-infectious inflammatory stress. Ahuja et al. [13] assessed immune resilience using two peripheral-blood metrics: immune health grades based on CD8^+^ and CD4^+^ T cell balance, and transcriptomic signatures reflecting survival-associated immunocompetence and mortality-associated inflammation. Across approximately 48,500 individuals, preservation of optimal immune resilience was associated with lower risk of HIV acquisition, AIDS progression, symptomatic influenza, recurrent skin cancer, survival during COVID-19 and sepsis, and longevity. This model is particularly relevant to exceptional longevity because it conceptualises healthy ageing as the maintenance of a favourable immunocompetence–inflammation balance rather than as the absence of inflammatory stress.

In a subsequent study, Manoharan et al. [158] linked immune resilience to a transcription factor 7 encoding T cell factor 1 (TCF7)-centred salutogenic programme, with TCF7 acting as a conserved regulator of T cell stemness and regenerative potential. Poor immune resilience was associated with a marked survival disadvantage, whereas optimal immune resilience preserved more favourable immune profiles, improved vaccine responses and reduced burdens of cardiovascular disease, Alzheimer’s disease and serious infections. Because TCF7 is present in all individuals, the relevant issue is not its presence or absence, but whether a TCF7-high immune transcriptional state is maintained or restored during ageing and inflammatory stress.

The accompanying editorial [159] further emphasised that TCF7-linked immune resilience may attenuate the pathogenic triad of inflammageing, immunosenescence and cellular senescence, thereby reframing healthy ageing as a salutogenic process rather than merely delayed pathology.

Together, these studies provide a useful framework for interpreting immune resilience as a dynamic property of ageing immune systems. In the context of exceptional longevity, they support the hypothesis that successful survival to very advanced age depends not on avoiding inflammatory stress, but on maintaining sufficient immune competence and restoring inflammatory control after repeated antigenic and environmental challenges. This perspective is highly consistent with the immune-inflammatory resilience observed in centenarians and in the oldest centenarians.

To explore how this immune balance translates into clinically relevant outcomes, we selected cancer and COVID-19 as two paradigmatic disease models: cancer, because it is a major age-associated disease in which immune surveillance, inflammation, cellular senescence and genomic maintenance are central, and COVID-19 because it exposed the vulnerability of the ageing immune system to novel infectious challenges while also highlighting the importance of immune-inflammatory resilience in very old individuals.

## 13. The Case of Cancer

### 13.1. Ageing as a Biological Context for Cancer Development

Cancer is one of the major age-associated diseases, together with cardiovascular, neurodegenerative, cognitive and metabolic disorders. Its incidence rises steeply with advancing age: the lifetime risk of developing cancer is relatively low before the age of 40, whereas it increases markedly in later life, reaching approximately 50% by 80 years of age. Accordingly, cancer is frequently regarded as a disease of ageing. In individuals over 60 years, the probability of developing cancer is more than twice that observed in younger adults, with diagnosis and cancer-related death occurring predominantly in later life [160,161].

The biological connections between ageing and cancer are increasingly being clarified. Both processes are driven, at least in part, by the progressive accumulation of cellular and molecular damage over time. Although cancer and ageing may appear to represent opposite cellular outcomes, uncontrolled proliferation and enhanced survival in cancer versus functional decline and reduced fitness in ageing, they share several mechanistic hallmarks. These include genomic instability, epigenetic remodelling, altered intercellular communication, impaired proteostasis, mitochondrial dysfunction, deregulated nutrient sensing pathway, telomere attrition and cellular senescence. As most cancers arise in aged tissues, many age-related alterations are already present in the cellular and tissue environment in which malignant transformation occurs. In this sense, a tissue enriched in damaged cells is simultaneously an ageing tissue and a tissue with increased susceptibility to cancer development. Ageing may promote tumorigenesis through multiple, interconnected pathways. The age-related accumulation of mutated clones, together with changes in the surrounding tissue environment, including stromal cell senescence, can favour clonal expansion and malignant progression. Tumour development usually requires genetic alterations that activate growth-promoting pathways, confer proliferative advantage and allow cells to bypass senescence or other tumour-suppressive barriers. Age-associated processes, such as DNA damage accumulation, telomere shortening, epigenetic alterations, proteostatic imbalance and metabolic dysregulation, may facilitate these events. In parallel, lifelong environmental exposures, including ultraviolet radiation, alcohol, tobacco smoke and pollution, further increase DNA damage and contribute to the molecular alterations associated with both ageing and carcinogenesis [160,161,162].

A key concept that has emerged in recent years is that ageing does not affect only potential cancer cells, but also profoundly remodels the normal cells of the tumour microenvironment. Fibroblasts, immune cells, endothelial cells and other stromal components may acquire age-related phenotypes that support tumour growth, immune escape, invasion and metastasis. Senescent stromal cells can produce inflammatory and tissue-remodelling mediators that alter local homeostasis and create conditions favourable to tumour progression. Thus, the relationship between ageing and cancer is not exclusively cell-autonomous; it is also shaped by the aged microenvironment, which can impose selective pressures that favour the expansion of pre-existing genetic or phenotypic variants within tissues. The relationship between ageing and cancer is therefore complex and, in some respects, paradoxical. The same processes that contribute to tissue degeneration, cellular senescence, apoptosis and structural decline may also create the conditions for malignant transformation in self-renewing tissues. Cancer risk and many degenerative features of tissues increase substantially with age, but their effects are not uniform across organs. Age-related reprogramming of local and systemic stromal environments may differ according to tissue type, thereby influencing tumour initiation, progression and metastatic behaviour in a cancer-specific manner. This helps explain why the association between age and clinical outcome varies among different malignancies [160].

### 13.2. Cancer Incidence and Behaviour at Extreme Ages

Several studies conducted in different geographical settings, including the USA, Europe, Japan, New Zealand and Brazil, indicate that both cancer incidence and cancer-related mortality decrease substantially at very advanced ages. Cancer rates appear to fall markedly after the age of 90, with reported values ranging from 0% to 4% among individuals aged 100 years and older. In addition, when cancer is diagnosed in centenarians, they often show a less aggressive clinical behaviour, with slower progression and a reduced tendency to metastasise [163,164]. This pattern is supported by analyses of global age-specific cancer mortality between 2000 and 2014. For most malignancies, except for prostate and breast cancer, mortality increased with age in both sexes but tended to slow down or reach a plateau after approximately 85 years. Among centenarians, cancer incidence often becomes extremely low, which may indicate either a decreased propensity to develop malignant disease or the emergence of a less aggressive tumour phenotype [165]. Similarly, data from the Surveillance, Epidemiology and End Results cancer registries, integrated with the 2000 US census, show that cancer incidence usually reaches its maximum between 75 and 90 years of age and then declines sharply in centenarians [166]. Autopsy studies from Japan provide further support for this observation. Compared with individuals aged 90–94 years, centenarians showed lower rates of metastatic disease and cancer-related death, with metastatic cancer reported in 37.5% versus 50.5% and cancer mortality in 16.7% versus 24.7%, respectively [167]. Similar results were obtained from Italian autopsies [168]. At the most extreme ages, supercentenarians may represent a particularly informative model of cancer resistance. In a study of supercentenarians from Okinawa, Japan, none had a clinical history of cancer [27]. However, another investigation describing the phenotypic characteristics of 32 age-validated supercentenarians reported that eight participants, corresponding to 25%, had a previous history of cancer. Importantly, all malignancies had been successfully treated, and none were active at the time of evaluation [26].

### 13.3. Mechanisms of Cancer Resistance in Exceptional Longevity

Several immune mechanisms may contribute to this discussed reduction in cancer burden at extreme ages. With regard to this, in the previous sections it has been suggested that extreme age is characterized by preserved NK cell activity, cytotoxic immune remodelling and a tightly regulated inflammatory tone [80]. This could support tumour immunosurveillance while limiting the chronic inflammation that favours tumour initiation and progression. Recent findings [127] described in Section 9 provide more specific support for a possible contribution of cytotoxic CD4^+^CTLs to cancer immunosurveillance in extreme longevity. In the follow-up study by Hashimoto et al., CD4^+^CTLs underwent an age-related expansion and formed large, clonally expanded populations without prominent signs of exhaustion. Unlike conventional cytotoxic CD8^+^ T cells, which recognize antigenic peptides presented by human leukocyte antigen (HLA) class I molecules, CD4^+^CTLs retain HLA class II-restricted antigen recognition while acquiring a cytotoxic effector programme. This feature may allow them to provide a complementary form of tumour immunosurveillance, particularly against MHC class II-expressing malignant cells. Interestingly, comparison of the CDR3β sequences of dominant CD4^+^CTL clones with a large public TCR database identified identical sequences among expanded T cell clones from patients with cancer, particularly in non-small cell lung cancer and adjacent tissues. Some sequences identified in healthy supercentenarians with no history of cancer matched those observed in patients with non-small cell lung cancer. These observations are consistent with the possibility that persistent antigen-driven CD4^+^CTL expansion may contribute to cancer surveillance at extreme ages. However, sequence identity alone does not establish antigen specificity, and the study therefore provides suggestive rather than direct evidence that these clones recognize cancer-associated antigens [127].

Notably, the decline in cancer mortality at extreme ages is not restricted to humans but has also been observed in some long-lived animal species. Genomic analyses of these species have identified distinctive adaptations in pathways involved in DNA repair, tumour suppression and immune regulation, suggesting that resistance to cancer may represent a conserved feature of exceptional survival. This is consistent with observations in human semi-supercentenarians and supercentenarians, in whom enhanced DNA repair capacity has been reported and may contribute to protection against the accumulation of genomic damage. Thus, reduced cancer burden at the extremes of lifespan may reflect, at least in part, the convergence of efficient genome maintenance, preserved tumour-suppressive mechanisms and effective immune surveillance [53,80]. It is intriguing that well-preserved p53-mediated responses have been suggested to be a factor contributing to protection from cancer in extreme longevity [169].

## 14. The Case of COVID-19

### 14.1. Age, Sex and COVID-19 Outcomes in the Oldest-Old

A recent narrative review revisiting the roles of age and sex in COVID-19 mortality has clearly shown that women generally display greater resistance to severe disease [70]. This finding is consistent with broader demographic evidence indicating that women tend to survive longer than men, even under highly adverse conditions such as famines and epidemics [170]. This does not exclude the possibility that, in specific settings, older women may be more severely affected than men, as it is well known that women live longer than men but are also more prone to disability in the final years of life [171]. Indeed, an Italian study analysed COVID-19-related vulnerability and mortality at the peak of the first wave of infection, in March 2020, in Lombardy, the region first affected in Europe. By using data on total deaths, rather than only confirmed COVID-19 cases, the authors concluded that excess mortality increased progressively up to very advanced ages. At the same time, however, men older than 90 years appeared to become relatively more resilient than age-matched women [172]. Consistent with this observation, a study conducted among residents of French nursing homes found substantially greater mortality in centenarians than in younger residents, despite a lower rate of hospitalisation [173]. In German long-term care facilities, centenarians also showed markedly reduced COVID-19-related hospital admission rates, but higher in-hospital mortality once admitted, again particularly among women [174].

Other population-based analyses, however, suggest a more complex scenario [80]. A Spanish study covering the period 2020–2022 found that, although documented COVID-19 cases were proportionally more frequent among centenarians than among non-centenarians, centenarians displayed prolonged survival after SARS-CoV-2 infection, with no intensive care unit admissions and survival curves more similar to those of 50-year-old individuals than to those of octogenarians or nonagenarians [175]. Similarly, a Colombian study reported better survival among centenarians than among individuals aged 80–99 years [176]. Japan represents an important exception to the general concern that COVID-19 disproportionately increased mortality among the oldest old. Indeed, during the pandemic, longevity among Japanese centenarians appears not to have declined. The projected number of centenarians rose from 37,005 in 2019 to 41,802 in the following year. At the same time, deaths from pneumonia between January and September 2020 decreased by 13,950 compared with the corresponding period of the previous year, while influenza deaths fell by 2340. This reduction in deaths from infectious diseases, which likely affected all older age groups, including centenarians, may reflect specific features of Japanese society [177]. Cultural practices may have contributed: traditional greetings in Japan do not usually involve handshaking, hugging or kissing, and mask-wearing was already common before the pandemic. In addition, Japan rapidly adopted the “3Cs” strategy, avoiding closed spaces, crowded places and close-contact settings, an approach that anticipated later scientific recognition of the importance of airborne SARS-CoV-2 transmission [178].

### 14.2. Birth Cohort Effects and the Spanish Flu Hypothesis

Although centenarians do not seem to experience lower mortality than other very old individuals, probably because of their high frailty burden, an intriguing pattern was observed during the first wave of the pandemic in 2020. Centenarians older than 101 years, born before 1919, but not relatively “younger” centenarians, appeared to display enhanced resilience to COVID-19 [179,180]. Indeed, in Belgian study, mortality was analysed according to year and month of birth among individuals who had reached 100 years of age during the COVID-19 pandemic and whose birth dates fell around the end of the First World War and the emergence of the H1N1 “Spanish flu” pandemic. COVID-19 mortality was significantly lower among “older” centenarians than among “younger” centenarians, with the strongest separation between the two groups observed when 1 August 1918 was used as the discriminating birthdate cut-off. After excluding the plausible influence of the end of the First World War, this date corresponded closely to the first reports of Spanish flu victims in Belgium. The temporal overlap between the onset of the 1918 influenza pandemic and the birth cohorts that later appeared more vulnerable to COVID-19 in 2020 suggested a possible relationship between early exposure to 1918 H1N1 influenza and subsequent resistance to SARS-CoV-2. One hypothesis was that long-lasting cross-reactive immune mechanisms may have enabled centenarians exposed to Spanish flu to better withstand COVID-19 a century later [179].

To explore this issue further, mortality data from Sicilian nonagenarians and centenarians during the first year of the COVID-19 pandemic, stratified by sex, was analysed using 2019 mortality data as the reference. Crude excess mortality was then calculated by comparing the two years. The data for Sicilian individuals aged 90 years and older indicate that, in line with known sex-related differences in responses to infection, the oldest women were more resilient to COVID-19 than men. Moreover, centenarians born before 1919, but not younger centenarians, appeared to be relatively protected against COVID-19-related mortality. Again, this observation might be linked to early-life exposure to the 1918 influenza pandemic [180].

### 14.3. Serological and Immune Evidence of COVID-19 Resilience in Oldest Centenarians

To investigate whether the ability of the oldest centenarians to tolerate SARS-CoV-2 infection might be linked to their historical exposure to the Spanish flu, a retrospective serological study was performed [132]. The study measured neutralising antibodies in serum samples from 33 centenarians, including 11 semi- and supercentenarians, born between 1905 and 1922, assessing their reactivity against both SARS-CoV-2 and the 1918 H1N1 influenza virus. Clinical records, together with laboratory evidence such as antibodies against the viral nucleocapsid protein, indicated that eight centenarians had experienced SARS-CoV-2 infection. Remarkably, despite the extreme age of three of these individuals, who were between 109 and 110 years old, their infections were asymptomatic or mild, and none required hospital admission. Of note, neutralising anti-spike antibody titres in infected or vaccinated centenarians were higher than those detected in a control group of randomly selected individuals aged approximately 70 years. These findings suggest that the oldest centenarians may possess an exceptional capacity to withstand COVID-19, combining mild or asymptomatic clinical courses with the ability to mount strong neutralising antibody responses. In addition, all centenarians showed markedly elevated antibody titres against the 1918 H1N1 virus (see below): centenarians whose blood samples had been collected before the COVID-19 pandemic were negative for SARS-CoV-2 antibodies but retained neutralising antibodies against the 1918 H1N1 virus, thereby excluding the possibility that the results were due to antibody cross-reactivity [132].

Consistent with these observations, another study described three Brazilian supercentenarians who survived SARS-CoV-2 infection in 2020, before vaccines became available. Despite their exceptional age, these individuals developed strong SARS-CoV-2-specific IgG and neutralising antibody responses, accompanied by enrichment of plasma proteins and metabolites related to innate immunity and host defence pathways [181]. Taken together, these data suggest that COVID-19 resilience in such individuals may arise from a favourable combination of genetic background and preserved functional competence of both innate and adaptive immune responses.

### 14.4. Immune-Inflammatory Resilience and Interpretation

Overall, the apparent resistance to COVID-19 observed among the oldest centenarians seems to be more closely associated with the effective control of immune-inflammatory responses, potentially distinguishing them from younger centenarians [93,149,150]. This does not exclude the possibility that previous H1N1 infection played a role by acting as a selective pressure, favouring the survival of individuals with a more robust immune system. However, in [34], the role of the complex interplay between sex, gender, age and the exposome in shaping responses to SARS-CoV-2 infection and vaccination is extensively discussed.

## 15. Discussion

The immune profile of centenarians and the oldest centenarians should not be regarded as the final stage of a linear immunological decline. Rather, it reflects a highly heterogeneous process of immune reorganisation in which age-related losses coexist with adaptive and compensatory responses. A central message is that immune ageing should be interpreted through the lens of immunobiography. Chronological age alone cannot account for the heterogeneity observed among older adults and centenarians, or even among the oldest centenarians themselves. Genetic background, sex, early-life exposures, nutrition, infections, vaccinations, the microbiota, environmental stressors, socioeconomic conditions and persistent viruses such as CMV all contribute to shaping individual immune trajectories. Accordingly, immune profiles associated with longevity in one individual or population may not be directly generalisable to others. Exceptional longevity may therefore be viewed as an ecological phenotype emerging from the lifelong interaction between biological resilience and the exposome.

Semi-supercentenarians and supercentenarians appear able to accommodate profound age-related immune changes while maintaining a functional balance among immune decline, memory, regulation and cytotoxic defence. The expansion of CD8^+^ terminally differentiated effector memory cells re-expressing CD45RA (αβ TEMRA cells), Vδ1^+^ TEMRA cells and NK cells, together with the enrichment of cytotoxic lymphocyte populations (CD4^+^CTLs) and the persistence of long-term immunological memory, may represent adaptive and compensatory mechanisms shaped by lifelong antigenic exposure. Preservation of NK-cell activity, cytotoxic T cell programmes, and durable immune memory may, in turn, contribute to the delay or attenuation of infections, cancer and other age-related diseases. Moreover, the oldest centenarians may display greater resistance to the detrimental consequences of inflammaging than older people and younger centenarians, potentially through several regulatory mechanisms involving miRNAs, cytokine networks and Tregs. Thus, some immune features conventionally regarded as manifestations of immunosenescence may, in the context of exceptional longevity, instead reflect successful adaptation to prolonged antigenic and inflammatory pressure.

Despite these advances, important questions remain, and several limitations warrant caution. Interpretation of the available evidence is constrained by small sample sizes, particularly in most transcriptomic studies, by differences in study design and analytical protocols, and by the predominance of European and East Asian cohorts. More importantly, and often overlooked, immunological studies of centenarians have relied predominantly on peripheral blood analyses, limiting the extent to which their findings can be extrapolated to tissues and organs. Although this limitation is understandable, the available evidence may not fully capture tissue-resident immune populations or local immune–stromal interactions. Furthermore, much of the evidence derives from cross-sectional comparisons, making it difficult to determine whether the observed immune features are causal determinants of survival or simply correlates of exceptional longevity. Longitudinal follow-up of centenarians as they transition to semi-supercentenarian and supercentenarian status could reveal additional adaptations required for survival beyond 100 years and provide further insight into the upper limits of the human lifespan.

The marked heterogeneity among centenarians also complicates the identification of universal biomarkers of healthy ageing and longevity. However, this heterogeneity is itself biologically meaningful, as it reflects the distinct immunobiography of each individual. Different biological trajectories may therefore converge on a state of relatively preserved immune competence without necessarily sharing the same cellular or molecular characteristics. Thus, extreme survival appears to reflect successful accommodation of age-related immune change through sustained cytotoxic surveillance, immune memory, inflammatory control and favourable lifelong adaptation to antigenic exposures.

## 16. Future Prospects

Future studies should integrate immunophenotyping, functional assays, cytokine and proteomic profiling, epigenetic and transcriptomic analyses, metabolic readouts, extracellular-vesicle studies and computational immune-age algorithms, ideally within harmonised longitudinal cohorts. Combining single-cell multi-omics approaches with longitudinal clinical data and environmental exposures, supported by artificial intelligence, will be essential to unravel the complexity of immune ageing. Insights gained from these exceptional individuals may help refine biomarkers of biological ageing, clarify the mechanisms underlying resilience and vulnerability, and guide the development of interventions aimed at preserving immune competence across the life course.

In conclusion, the global population continues to age, translating these insights into strategies capable of extending healthspan will remain a central objective of ageing research.

## Figures and Tables

**Table 1:** **Categories of exceptional longevity and their immunological relevance**. Exceptional longevity includes biologically distinct categories, ranging from long-living individuals (LLIs) to centenarians, semi-supercentenarians and supercentenarians. These groups differ not only in chronological age and demographic structure, but also in the degree of biological selection, compression of morbidity and immune adaptation.

Category	Age Range	Demographic Features	Clinical Relevance	Immune Features	Main Limitations of Available Studies	Ref.
LLIs	Usually ≥90 years.	Female predominance less marked than in centenarians. Heterogeneous in health status and lifestyle.	Useful for identifying early markers of healthy (or unhealthy) ageing before the centenarian threshold.	Immune-inflammatory profiles are highly variable and strongly shaped by immunobiography.	Heterogeneous definitions and cohort heterogeneity in terms of frailty and disease burden.	[16,17,18,19,20]
Centenarians	≥100 years	Strong female predominance. Considerable heterogeneity in health status.	Model for studying survival to 100 years, delayed morbidity.	Reduced naïve T cell output, expansion of memory and differentiated T cells, changes in B cell compartments, increased NK cell proportions in some cohorts, and variable inflammaging.	Cross-sectional designs; different recruitment criteria and variable health status. Female predominance makes sex-specific analyses difficult.	[1,2,17,18,19,20,21,22]
Semi-supercentenarians	105–109 years	Very rare; marked female predominance.	Model of transition from exceptional to extreme longevity, including disease resistance and delayed disability.	Preserved immune memory, cytotoxic remodelling, natural killer (NK) cell expansion, αβ and γ/δ T cell changes and better regulation of inflammation. controlled than expected for age.	Very small cohorts, scarce males. Results may be influenced by population-specific factors.	[23,24,25,26]
Supercentenarians	≥10 years	Extremely rare; overwhelming female predominance.	Model to investigate the limits of human lifespan and of compression of morbidity.,	Preserved cytotoxic surveillance, adaptive expansion of NK and cytotoxic T cell compartments, long-term immune memory, controlled inflammatory responses.	Extremely small samples; mainly descriptive data; poor generalisability.	[26,27,28,29]

Exceptional longevity encompasses biologically distinct categories, ranging from long-lived individuals to centenarians, semi-supercentenarians, and supercentenarians (the latter two groups are also referred to as oldest centenarians in this review). These groups differ not only in chronological age and demographic structure, but also in the degree of biological selection, compression of morbidity and immune adaptation. Thus, the immunological profile of exceptional longevity should not be interpreted as a single phenotype, but as a continuum in which immune ageing, compensatory remodelling, inflammatory regulation and lifelong antigenic experience acquire different meanings as survival extends from old age to the most extreme limits of human lifespan (see next sections).

**Table 2:** **Single-cell transcriptomic evidence of T and B cell remodelling in exceptional longevity.**

Authors, Year	Population/Approach	T Cell Findings	B Cell Findings	Interpretation	Ref.
Hashimoto et al. 2019	7 supercentenarians, 5 younger controls; scRNA-seq with TCR analysis.	Marked expansion of clonally expanded cytotoxic CD4^+^ T cells with CD8-like effector features.	B cell data were limited. Naïve B cells were significantly lower in supercentenarians.	Extreme longevity was mainly associated with cytotoxic CD4^+^ T cell remodelling.	[126]
Dong et al. 2022	Chinese centenarians, their offspring and controls; scRNA-seq.	Expansion and functional reinforcement of cytotoxic T cell populations.	B cell results were secondary in this analysis.	Familial and exceptional longevity were associated mainly with reinforced cytotoxic T cell programmes.	[131]
Karagiannis et al. 2023	7 centenarians (mean age 106 years), integrated with public datasets.	Age-related shifts from lymphoid to myeloid cells and from non-cytotoxic to cytotoxic cells.	Distinctive redistribution from CD4^+^ T cells towards B cell populations.	Extensive immune remodelling rather than preservation of a youthful profile.	[130]
Wang et al. 2025	7 supercentenarians, 3 centenarians, 4 older controls; 87,215 PBMCs, scRNA-seq + FACS.	Enrichment of CD8^+^ effector-memory T cells and CD4^+^ cytotoxic T cells.	Enrichment of memory B cells, plasma cells and terminally differentiated B-cell states. Naïve B cells less represented.	Coordinated T and B cell remodelling may support preserved immune competence.	[129]
Hashimoto et al. 2026	8 individuals aged 70–99 years, 10 centenarians and 10 supercentenarians; scRNA-seq), surface-protein and TCR profiling.	Progressive expansion of CD4^+^CTLs; sequential CD27/CD28 loss; large private clones; preserved cytotoxic programme without prominent exhaustion; cytokine plasticity.	Not assessed; study focused on T cells.	CD4^+^CTL expansion may represent adaptive clonal remodelling in response to persistent antigenic stimulation.	[127]

**Table 3:** **Protective versus potentially detrimental immune features in exceptional longevity.**

Immune Feature	Potentially Protective Interpretation	Potentially Detrimental Interpretation	Relevance to Exceptional Longevity	Ref.
**Myeloid bias**	Supports innate immune readiness and rapid responses to injury or infection.	May promote systemic inflammation, monocyte activation, and impaired adaptive immunity.	Compatible with exceptional longevity only if inflammatory activation is controlled.	[59,60,61]
**NK cell expansion**	Enhances cancer immunosurveillance and antiviral defence.	May accompany immune dysregulation or chronic tissue-damaging inflammation.	May represent compensatory cytotoxic remodelling rather than immune decline.	[80,96,97,98,99]
**Reduced naïve T cell compartment**	May be partly compensated by peripheral homeostasis and memory-cell persistence.	Reduces TCR diversity and responses to novel antigens.	Their impact may be mitigated by effective immune memory and regulatory balance.	[12,76,80,97,107,108,126,129,130]
**CMV-driven immune remodelling**	Promotes expansion of memory-like cytotoxic T- and NK cell populations.	May expand terminally differentiated T cells and increase inflammatory burden.	Effects are context-dependent and shaped by immunobiography.	[12,81,96,100,102,108]
**Expansion of CD8^+^ TEMRA cells**	Maintains cytotoxic memory against lifelong antigenic exposures (as CMV).	May reflect chronic antigenic load, clonal restriction and immune exhaustion.	Potentially adaptive when contributing to immune memory and cytotoxic defence, without excessive clonal restriction or inflammatory burden.	[12,76,80,97,107,108]
**Cytotoxic surveillance**	Eliminates infected, transformed and senescent cells.	Excessive cytotoxicity may contribute to tissue injury.	Preserved cytotoxic surveillance, particularly through NK cells and cytotoxic T cells (including CD4^+^CTLs), may be one of the adaptive immune traits of extreme longevity.	[126,127,129,131]
**γδ T cell remodelling**	Supports barrier surveillance, stress responses and cancer recognition.	Persistent activation may contribute to chronic inflammation.	May form part of a broader compensatory immune architecture shaped by immunobiography.	[109,110,111,112].
**Regulatory immune activity**	Treg cells and anti-inflammatory pathways may limit tissue damage and restrain inflammaging.	Excessive regulation may impair pathogen and cancer control.	Balanced regulation can prevent chronic inflammation without suppressing protective immunity.	[80,107,146,147,148,149,150]
**Preserved immune memory**	May maintain protection despite reduced naïve-cell output.	Excessive expansion of memory compartments may limit responses to novel antigens.	Healthy ageing may require durable memory together with sufficient immune flexibility.	[132,143]
**Inflammaging**	A low or well-regulated inflammation may support host defence and immune surveillance.	Persistent systemic inflammation promotes frailty, neurodegeneration, cancer and mortality.	Longevity may depend more on regulation than on absence of inflammaging.	[11,13,80,81,83,87,88,89,147,149,150]
**Immunobiography-shaped adaptation**	Lifelong exposure to antigens may generate a personalised immune architecture.	Unfavourable exposures may drive chronic inflammation, immune exhaustion, and clonal restriction.	A favourable immunobiographical trajectory may help maintain the immune system in functional equilibrium.	[11,12,81]

These immune features should not be classified rigidly as beneficial or harmful. In exceptional longevity, their biological meaning depends on context, intensity, timing and regulation. Traits traditionally viewed as markers of immunosenescence may be compatible with healthy survival when embedded in a balanced system characterised by preserved cytotoxic surveillance, controlled inflammaging, effective immune memory and regulatory restraint. References in the text.

## Data Availability

No new data were created or analysed in this study. Data sharing is not applicable to this article.

## Abbreviations

The following abbreviations are used in this manuscript:

ABCs	Age-associated B cells
AID	Activation-induced cytidine deaminase
BCR	B cell receptor
Casin	Cdc42 activity-specific inhibitor
Cdc42	Cell division cycle 42
CHIP	Clonal haematopoiesis of indeterminate potential
CMV	Cytomegalovirus
CRP	C reactive protein
DC	Dendritic cell
DN	Double negative
ECs	Senescent endothelial cells
FACS	Fluorescence-activated cell sorting
FCRL 2	Fc receptor-like 2
GZM	Granzyme
GWAS	Genome-wide association studies
HLA	Human leukocyte antigen
HSCs	Human haematopoietic stem cells
IFN	Interferon
IL	Interleukin
IL-7R	IL-7 receptor
KLRG1	Killer cell lectin-like receptor subfamily G member 1
LLIs	Long-living individuals
miRNA	Micro RNA
NK	Natural killer
NLRP3	NOD-like receptor family pyrin domain-containing protein 3
PBMC	Peripheral blood mononuclear cells
SASP	Senescence-associated secretory phenotype
SCFAs	Short-chain fatty acids
SIRI	Systemic inflammation response index
SOX	SRY-box transcription factor
TCF7	Transcription factor 7 encoding T cell factor 1
TCR	T cell receptor
TEMRA	T effector memory cells re-expressing CD45RA
TLR	Toll-like receptor
Treg	T regulatory cell
XCI	X-chromosome inactivation

## References

[1] Accardi G., Aiello A., Vasto S., Caruso C., Caruso C. (2019). Chance and Causality in Ageing and Longevity. Centenarians.

[2] Accardi G., Ligotti M.E., Candore G., Caruso C. (2019). Phenotypic Aspects of Longevity. Centenarians.

[3] Finch C.E., Haghani A. (2021). Gene-Environment Interactions and Stochastic Variations in the Gero-Exposome. J. Gerontol. A Biol. Sci. Med. Sci..

[4] Shenhar B., Pridham G., De Oliveira T.L., Raz N., Yang Y., Deelen J., Hägg S., Alon U. (2026). Heritability of intrinsic human life span is about 50% when confounding factors are addressed. Science.

[5] Aliberti S.M., Capunzo M., Galimberti D., Accardi G., Aiello A., Calabrò A., Caruso C., Candore G. (2025). Ageing Trajectories: Exposome-Driven Pathobiological Mechanisms and Implications for Prevention from Blue Zones and Italian Longevity Hotspots Such as Cilento and Sicilian Mountain Villages. Int. J. Mol. Sci..

[6] Aliberti S.M., Capunzo M. (2025). The Power of Environment: A Comprehensive Review of the Exposome’s Role in Healthy Aging, Longevity, and Preventive Medicine-Lessons from Blue Zones and Cilento. Nutrients.

[7] López-Otín C., Kroemer G. (2025). Hallmarks of aging: Integrating molecular and social determinants. Geromedicine.

[8] Bradley E., Haran J. (2024). The human gut microbiome and aging. Gut Microbes.

[9] Kim M., Benayoun B.A. (2020). The microbiome: An emerging key player in aging and longevity. Transl. Med. Aging.

[10] Candore G., Aiello A., Caruso C., Caruso C. (2025). Role of sex and gender in aging and longevity: An introduction. Role of Sex and Gender in Aging and Longevity.

[11] Franceschi C., Salvioli S., Garagnani P., de Eguileor M., Monti D., Capri M. (2017). Immunobiography and the Heterogeneity of Immune Responses in the Elderly: A Focus on Inflammaging and Trained Immunity. Front. Immunol..

[12] Caruso C., Ligotti M.E., Accardi G., Aiello A., Candore G. (2022). An immunologist’s guide to immunosenescence and its treatment. Expert Rev. Clin. Immunol..

[13] Ahuja S.K., Manoharan M.S., Lee G.C., McKinnon L.R., Meunier J.A., Steri M. (2023). Immune resilience despite inflammatory stress promotes longevity and favorable health outcomes including resistance to infection. Nat. Commun..

[14] Italian National Institute of Statistics (ISTAT). https://www.istat.it/comunicato-stampa/i-centenari-in-italia-al-1-gennaio-2025/.

[15] The Supercentenari d’Italia Database of the Oldest Living People in Italy. https://www.supercentenariditalia.it/persone-viventi-piu-longeve-in-italia.

[16] Aiello A., Accardi G., Aprile S., Caldarella R., Carru C., Ciaccio M., De Vivo I., Gambino C.M., Ligotti M.E., Vasto S. (2021). Age and Gender-related Variations of Molecular and Phenotypic Parameters in A Cohort of Sicilian Population: From Young to Centenarians. Aging Dis..

[17] Arai Y., Sasaki T., Hirose N. (2017). Demographic, phenotypic, and genetic characteristics of centenarians in Okinawa and Honshu, Japan: Part 2 Honshu, Japan. Mech. Ageing Dev..

[18] Caballero Ó., García-Hermoso A., Hurtado-Almonacid J., Yáñez-Sepúlveda R., López-Gil J.F., Ezzatvar Y. (2026). Lifestyle, socioeconomic, and biological determinants of survival in nonagenarians and centenarians: A systematic review and meta-analysis. Gerontologist.

[19] Candal-Pedreira C., Rey-Brandariz J., Martín-Gisbert L., Teijeiro A., García G., Ruano-Ravina A., Pérez-Ríos M. (2025). Blue Zones, an Analysis of Existing Evidence through a Scoping Review. Aging Dis..

[20] Dai Z., Lee S.Y., Sharma S., Ullah S., Tan E.C.K., Brodaty H., Schutte A.E., Sachdev P.S. (2024). A systematic review of diet and medication use among centenarians and near-centenarians worldwide. Geroscience.

[21] Pignolo R.J. (2019). Exceptional Human Longevity. Mayo Clin. Proc..

[22] Vetrani C., Frias-Toral E., Annunziata G., Amatrudo F., Mayol D., Suárez R., Verde L., Negri M., Piscitelli P., Colao A. (2026). Longevity, Centenarians, and Lifestyle: Any ’’Tips’’ to Live Longer?. Curr. Nutr. Rep..

[23] Accardi G., Aiello A., Aprile S., Calabrò A., Caldarella R., Caruso C., Ciaccio M., Dieli F., Ligotti M.E., Meraviglia S. (2023). The Phenotypic Characterization of the Oldest Italian Man from December 28, 2020, to September 23, 2021, A.T., Strengthens the Idea That the Immune System can Play a Key Role in the Attainment of Extreme Longevity. J. Clin. Med..

[24] Accardi G., Aiello A., Aprile S., Caldarella R., Cammarata G., Carru C., Caruso C., Ciaccio M., Colomba P., Galimberti D. (2020). The Phenotypic Characterization of the Cammalleri Sisters, an Example of Exceptional Longevity. Rejuvenation Res..

[25] Arai Y., Martin-Ruiz C.M., Takayama M., Abe Y., Takebayashi T., Koyasu S., Suematsu M., Hirose N., von Zglinicki T. (2015). Inflammation, But Not Telomere Length, Predicts Successful Ageing at Extreme Old Age: A Longitudinal Study of Semi-supercentenarians. EBioMedicine.

[26] Abdelraheem O., El Kheir-Mataria W.A., Chun S. (2026). Genetic, Socioecological, and Health Research on Extreme Longevity in Semisupercentenarians and Supercentenarians: A Scoping Review. J. Aging Res..

[27] Andersen S.L., Sebastiani P., Dworkis D.A., Feldman L., Perls T.T. (2012). Health span approximates life span among many supercentenarians: Compression of morbidity at the approximate limit of life span. J. Gerontol. A Biol. Sci. Med. Sci..

[28] Schoenhofen E.A., Wyszynski D.F., Andersen S., Pennington J., Young R., Terry D.F., Perls T.T. (2006). Characteristics of 32 supercentenarians. J. Am. Geriatr. Soc..

[29] Willcox D.C., Willcox B.J., Wang N.C., He Q., Rosenbaum M., Suzuki M. (2008). Life at the extreme limit: Phenotypic characteristics of supercentenarians in Okinawa. J. Gerontol. A Biol. Sci. Med. Sci..

[30] Staerk J., Conde D.A., Tidière M., Lemaître J.F., Liker A., Vági B., Pavard S., Giraudeau M., Smeele S.Q., Vincze O. (2025). Sexual selection drives sex difference in adult life expectancy across mammals and birds. Sci. Adv..

[31] Sun Z., Fan J., Wang Y. (2022). X-Chromosome Inactivation and Related Diseases. Genet Res..

[32] Caruso C., Accardi G., Aiello A., Candore G., Kaye P.M. (2025). Immunity and Ageing. Encyclopedia of Immunobiology.

[33] Calabrò A., Aiello A., Montomoli E., Pawelec G., Trombetta C.M., Caruso C. (2026). The influence of sex and gender differences in shaping the immune response to influenza infection and vaccination. Explor. Immunol..

[34] Calabrò A., Accardi G., Montomoli E., Pawelec G., Trombetta C.M., Caruso C. (2026). COVID-19, Immunity, and Vaccination: Unravelling the Interplay of Sex, Gender, Age, and the Exposome. Explor. Immunol..

[35] Feng Z., Liao M., Zhang L. (2024). Sex differences in disease: Sex chromosome and immunity. J. Transl. Med..

[36] Laffont S., Seillet C., Guéry J.C. (2017). Estrogen Receptor-Dependent Regulation of Dendritic Cell Development and Function. Front. Immunol..

[37] Layug P.J., Vats H., Kannan K., Arsenio J. (2024). Sex differences in CD8^+^ T cell responses during adaptive immunity. WIREs Mech. Dis..

[38] Peckham H., Radziszewska A., Sikora J., de Gruijter N.M., Restuadi R., Kartawinata M., Martin-Gutierrez L., Robinson G.A., Deakin C.T., Wedderburn L.R. (2025). Estrogen influences class-switched memory B cell frequency only in humans with two X chromosomes. J. Exp. Med..

[39] Kirkwood T.B. (1977). Evolution of ageing. Nature.

[40] Hayflick L. (2007). Entropy explains aging, genetic determinism explains longevity, and undefined terminology explains misunderstanding both. PLoS Genet..

[41] Cho M., Suh Y. (2014). Genome maintenance and human longevity. Curr. Opin. Genet. Dev..

[42] Caruso C., Ligotti M.E., Accardi G., Aiello A., Duro G., Galimberti D., Candore G. (2022). How Important Are Genes to Achieve Longevity?. Int. J. Mol. Sci..

[43] Montesanto A., Latorre V., Giordano M., Martino C., Domma F., Passarino G. (2011). The genetic component of human longevity: Analysis of the survival advantage of parents and siblings of Italian nonagenarians. Eur. J. Hum. Genet..

[44] Herskind A.M., McGue M., Holm N.V., Sørensen T.I., Harvald B., Vaupel J.W. (1996). The heritability of human longevity: A population-based study of 2872 Danish twin pairs born 1870–1900. Hum. Genet..

[45] Skytthe A., Pedersen N.L., Kaprio J., Stazi M.A., Hjelmborg J.V., Iachine I. (2003). Longevity studies in GenomEUtwin. Twin Res..

[46] VB Hjelmborg J., Iachine I., Skytthe A., Vaupel J.W., McGue M., Koskenvuo M., Kaprio J., Pedersen N.L., Christensen K. (2006). Genetic influence on human lifespan and longevity. Hum. Genet..

[47] Brooks-Wilson A.R. (2013). Genetics of healthy aging and longevity. Hum. Genet..

[48] Sebastiani P., Solovieff N., Dewan A.T., Walsh K.M., Puca A., Hartley S.W., Melista E., Andersen S., Dworkis D.A., Wilk J.B. (2012). Genetic signatures of exceptional longevity in humans. PLoS ONE.

[49] Sebastiani P., Gurinovich A., Bae H., Andersen S., Malovini A., Atzmon G., Villa F., Kraja A.T., Ben-Avraham D., Barzilai N. (2017). Four Genome-Wide Association Studies Identify New Extreme Longevity Variants. J. Gerontol. A Biol. Sci. Med. Sci..

[50] Revelas M., Thalamuthu A., Oldmeadow C., Evans T.J., Armstrong N.J., Kwok J.B., Brodaty H., Schofield P.R., Scott R.J., Sachdev P.S. (2018). Review and meta-analysis of genetic polymorphisms associated with exceptional human longevity. Mech. Ageing Dev..

[51] Boyce W.T., Sokolowski M.B., Robinson G.E. (2020). Genes and environments, development and time. Proc. Natl. Acad. Sci. USA.

[52] World Health Organization (WHO). https://www.who.int/health-topics/cardiovascular-diseases#tab=tab_1.

[53] Garagnani P., Marquis J., Delledonne M., Pirazzini C., Marasco E., Kwiatkowska K.M., Iannuzzi V., Bacalini M.G., Valsesia A., Carayol J. (2021). Whole-genome sequencing analysis of semi-supercentenarians. eLife.

[54] Sebastiani P., Perls T.T. (2012). The genetics of extreme longevity: Lessons from the new England centenarian study. Front. Genet..

[55] Gurinovich A., Andersen S.L., Puca A., Atzmon G., Barzilai N., Sebastiani P. (2019). Varying Effects of APOE Alleles on Extreme Longevity in European Ethnicities. J. Gerontol. A Biol. Sci. Med. Sci..

[56] Giuliani C., Garagnani P., Franceschi C. (2018). Genetics of Human Longevity Within an Eco-Evolutionary Nature-Nurture Framework. Circ. Res..

[57] Gavrilov L.A., Gavrilova N.S. (2011). Season of birth and exceptional longevity: Comparative study of American centenarians, their siblings, and spouses. J. Aging Res..

[58] Poulain M., Herm A. (2025). Season of Birth and Survival to 105 Years: Demographic Evidence. Ageing Longev. Res..

[59] Bagnara G.P., Bonsi L., Strippoli P., Bonifazi F., Tonelli R., D’Addato S., Paganelli R., Scala E., Fagiolo U., Monti D. (2000). Hemopoiesis in healthy old people and centenarians: Well-maintained responsiveness of CD34^+^ cells to hemopoietic growth factors and remodeling of cytokine network. J. Gerontol. A Biol. Sci. Med. Sci..

[60] Ross J.B., Myers L.M., Noh J.J., Collins M.M., Carmody A.B., Messer R.J., Dhuey E., Hasenkrug K.J., Weissman I.L. (2024). Depleting myeloid-biased haematopoietic stem cells rejuvenates aged immunity. Nature.

[61] Abbott A. (2024). Hacking the immune system could slow ageing-here’s how. Nature.

[62] Florian M.C., Klose M., Sacma M., Jablanovic J., Knudson L., Nattamai K.J., Marka G., Vollmer A., Soller K., Sakk V. (2018). Aging alters the epigenetic asymmetry of HSC division. PLoS Biol..

[63] Florian M.C., Leins H., Gobs M., Han Y., Marka G., Soller K., Vollmer A., Sakk V., Nattamai K.J., Rayes A. (2020). Inhibition of Cdc42 activity extends lifespan and decreases circulating inflammatory cytokines in aged female C57BL/6 mice. Aging Cell.

[64] Montserrat-Vazquez S., Ali N.J., Matteini F., Lozano J., Zhaowei T., Mejia-Ramirez E., Marka G., Vollmer A., Soller K., Sacma M. (2022). Transplanting rejuvenated blood stem cells extends lifespan of aged, immunocompromised mice. npj Regen. Med..

[65] Jaiswal S., Ebert B.L. (2019). Clonal hematopoiesis in human aging and disease. Science.

[66] Bains I., Thiébaut R., Yates A.J., Callard R. (2009). Quantifying thymic export: Combining models of naive T cell proliferation and TCR excision circle dynamics gives an explicit measure of thymic output. J. Immunol..

[67] Miller J.F. (1961). Immunological function of the thymus. Lancet.

[68] Sutherland J.S., Goldberg G.L., Hammett M.V., Uldrich A.P., Berzins S.P., Heng T.S., Blazar B.R., Millar J.L., A Malin M., Chidgey A.P. (2005). Activation of thymic regeneration in mice and humans following androgen blockade. J. Immunol..

[69] Nasi M., Troiano L., Lugli E., Pinti M., Ferraresi R., Monterastelli E., Mussi C., Salvioli G., Franceschi C., Cossarizza A. (2006). Thymic output and functionality of the IL-7/IL-7 receptor system in centenarians: Implications for the neolymphogenesis at the limit of human life. Aging Cell.

[70] Caruso C., Marcon G., Accardi G., Aiello A., Calabrò A., Ligotti M.E., Tettamanti M., Franceschi C., Candore G. (2023). Role of Sex and Age in Fatal Outcomes of COVID-19: Women and Older Centenarians Are More Resilient. Int. J. Mol. Sci..

[71] Bernatz S., Prudente V., Pai S., Attermann A.K., Cao Y., Chen J., Lyass A., Foldyna B., Nürnberg L., Bressem K. (2026). Thymic health consequences in adults. Nature.

[72] Bernatz S., Prudente V., Pai S., Attermann A.K., Di Federico A., Rowan A., Veeriah S., Dyrskjøt L., Nürnberg L., Alessi J.V. (2026). Thymic health and immunotherapy outcomes in patients with cancer. Nature.

[73] Chen E. (2026). Could this mysterious disappearing organ hold the key to longevity?. Nature.

[74] Kooshesh K.A., Foy B.H., Sykes D.B., Gustafsson K., Scadden D.T. (2023). Health Consequences of Thymus Removal in Adults. N. Engl. J. Med..

[75] Resio B.J., Canavan M., Caturegli G., Detterbeck F., Boffa D.J. (2026). Mortality Risk and Cause of Death Associated With Removal of the Adult Thymus: Analysis of the U.S. Thymoma Population. J. Thorac. Oncol..

[76] Calabrò A., Accardi G., Aiello A., Caruso C., Candore G. (2023). Sex and gender affect immune aging. Front. Aging.

[77] Moskalev A., Stambler I., Caruso C. (2020). Innate and Adaptive Immunity in Aging and Longevity: The Foundation of Resilience. Aging Dis..

[78] Jang I.H., Niedernhofer L.J., Robbins P.D., Camell C.D. (2026). The ageing immune system as a driver of systemic ageing. Nat. Rev. Immunol..

[79] Aiello A., Ligotti M.E., Garnica M., Accardi G., Calabrò A., Pojero F., Arasanz H., Bocanegra A., Blanco E., Chocarro L. (2022). How Can We Improve Vaccination Response in Old People? Part I: Targeting Immunosenescence of Innate Immunity Cells. Int. J. Mol. Sci..

[80] Plaza-Florido A., Carrera-Bastos P., Pérez-Prieto I., Fiuza-Luces C., Radom-Aizik S., Del Pozo Cruz B., Franceschi C., López-Soto A., López-Otín C., Lucia A. (2026). The long-lived immune system of centenarians. Nat. Rev. Immunol..

[81] Cossarizza A., Poli G., Clerici M. (2026). The silent infection load: How lifelong asymptomatic infections may contribute to inflammaging as an evolutionary trade-off of longevity. Ageing Res. Rev..

[82] Anaya J.M., Lozada-Martinez I.D., Acosta-Ampudia Y., Tobón G. (2026). Immunosenescence and human healthspan. Lessons from centenarians. Curr. Opin. Immunol..

[83] Wrona M.V., Ghosh R., Coll K., Chun C., Yousefzadeh M.J. (2024). The 3 I’s of immunity and aging: Immunosenescence, inflammaging, and immune resilience. Front. Aging.

[84] Sopena-Rios M., Ripoll-Cladellas A., Omidi F., Ballouz S., Alquicira-Hernandez J., Oelen R., Hewitt A.W., Franke L., van der Wijst M.G.P., Powell J.E. (2026). Single-cell analysis of the human immune system reveals sex-specific dynamics of immunosenescence. Nat. Aging.

[85] Hirokawa K., Utsuyama M., Hayashi Y., Kitagawa M., Makinodan T., Fulop T. (2013). Slower immune system aging in women versus men in the Japanese population. Immun. Ageing.

[86] Márquez E.J., Chung C.H., Marches R., Rossi R.J., Nehar-Belaid D., Eroglu A., Mellert D.J., Kuchel G.A., Banchereau J., Ucar D. (2020). Sexual-dimorphism in human immune system aging. Nat. Commun..

[87] Bonafè M., Olivieri F. (2022). Inflammaging: The lesson of COVID-19 pandemic. Mech. Ageing Dev..

[88] Karpuzoglu E., Holladay S.D., Gogal R.M. (2025). Inflammaging: Triggers, molecular mechanisms, immunological consequences, sex differences, and cutaneous manifestations. Front. Immunol..

[89] Caldarelli M., Rio P., Marrone A., Giambra V., Gasbarrini A., Gambassi G., Cianci R. (2024). Inflammaging: The Next Challenge-Exploring the Role of Gut Microbiota, Environmental Factors, and Sex Differences. Biomedicines.

[90] Dominguez L.J., Veronese N., Barbagallo M. (2024). Dietary Patterns and Healthy or Unhealthy Aging. Gerontology.

[91] Liu X.F., Shao J.H., Liao Y.T., Wang L.N., Jia Y., Dong P.J., Liu Z.Z., He D.D., Li C., Zhang X. (2023). Regulation of short-chain fatty acids in the immune system. Front. Immunol..

[92] Vishal V., Sahoo S., Lenka P., Ghosh S. (2025). A report on the gut bacterial biodiversity of a centenarian and supercentenarian lacto-vegetarian Indian females. Microbe.

[93] Santos-Pujol E., Noguera-Castells A., Casado-Pelaez M., García-Prieto C.A., Vasallo C., Campillo-Marcos I., Quero-Dotor C., Crespo-García E., Bueno-Costa A., Setién F. (2025). The multiomics blueprint of the individual with the most extreme lifespan. Cell Rep. Med..

[94] Valeri F., Endres K. (2021). How biological sex of the host shapes its gut microbiota. Front. Neuroendocrinol..

[95] Yoon K., Kim N. (2021). Roles of sex hormones and gender in the gut microbiota. J. Neurogastroenterol. Motil..

[96] Campos C., Pera A., Pita-Lopez M.L., Lopez-Cejas N., Hassouneh F., Sánchez-Correa B., Gayoso I., Alonso C., Peralbo E., Casado J.G., Fulop T., Franceschi C., Hirokawa K., Pawelec G. (2019). Natural Killer Cells in Human Aging. Handbook of Immunosenescence.

[97] Ligotti M.E., Aiello A., Accardi G., Aprile S., Bonura F., Bulati M., Gervasi F., Giammanco G.M., Pojero F., Zareian N. (2021). Analysis of T and NK cell subsets in the Sicilian population from young to supercentenarian: The role of age and gender. Clin. Exp. Immunol..

[98] Ligotti M.E., Accardi G., Aiello A., Calabrò A., Caruso C., Corsale A.M., Dieli F., Di Simone M., Meraviglia S., Candore G. (2023). Sicilian Semi- and Supercentenarians: Age-related NK Cell Immunophenotype and Longevity Trait Definition. Transl. Med. UniSa.

[99] Bertolazzi G., Calabrò A., Accardi G., Aiello A., Caruso C., Corsale A.M., Di Simonet M., Caldarella R., Meraviglia S., Candore G. (2026). Beyond chronological age: Sex and IL-6 are associated with natural killer cell and monocyte counts in a Sicilian cohort spanning adulthood to extreme longevity. Geromedicine.

[100] Bertolazzi G., Calabrò A., Accardi G., Aiello A., Caruso C., Corsale A.M., Di Simonet M., Meraviglia S., Candore G. (2026). B Cell Levels in Centenarians, Semi-Supercentenarians, and Supercentenarians: Descriptive Analysis by Age, Sex, Cytomegalovirus Status, and Interleukin-6. J. Ageing Longev..

[101] Cheng M.I., Li J.H., Riggan L., Chen B., Tafti R.Y., Chin S., Ma F., Pellegrini M., Hrncir H., Arnold A.P. (2023). The X-linked epigenetic regulator UTX controls NK cell-intrinsic sex differences. Nat. Immunol..

[102] Solana R., Campos C., Pera A., Tarazona R. (2014). Shaping of NK cell subsets by aging. Curr. Opin. Immunol..

[103] Pinti M., De Biasi S., Gibellini L., Lo Tartaro D., De Gaetano A., Mattioli M., Caruso C., Candore G. (2021). Aging of immune system. Human Aging.

[104] Mittelbrunn M., Kroemer G. (2021). Hallmarks of T cell aging. Nat. Immunol..

[105] Liu Z., Liang Q., Ren Y., Guo C., Ge X., Wang L., Cheng Q., Luo P., Zhang Y., Han X. (2023). Immunosenescence: Molecular mechanisms and diseases. Signal Transduct. Target. Ther..

[106] Goronzy J.J., Weyand C.M. (2019). Mechanisms underlying T cell ageing. Nat. Rev. Immunol..

[107] Bianchini E., Pecorini S., De Biasi S., Gibellini L., Nasi M., Cossarizza A., Pinti M., Fulop T., Franceschi C., Hirokawa K., Pawelec G. (2019). Lymphocyte Subtypes and Functions in Centenarians as Models for Successful Aging. Handbook of Immunosenescence.

[108] Ligotti M.E., Accardi G., Aiello A., Aprile S., Calabrò A., Caldarella R., Caruso C., Ciaccio M., Corsale A.M., Dieli F. (2023). Sicilian semi- and supercentenarians: Identification of age-related T-cell immunophenotype to define longevity trait. Clin. Exp. Immunol..

[109] Argentati K., Re F., Donnini A., Tucci M.G., Franceschi C., Bartozzi B., Betrnardini G., Provinciali M. (2002). Numerical and functional alterations of circulating gammadelta T lymphocytes in aged people and centenarians. J. Leukoc. Biol..

[110] Colonna-Romano G., Potestio M., Aquino A., Candore G., Lio D., Caruso C. (2002). Gamma/delta T lymphocytes are affected in the elderly. Exp. Gerontol..

[111] Colonna-Romano G., Aquino A., Bulati M., Lio D., Candore G., Oddo G., Scialabba G., Vitello S., Caruso C. (2004). Impairment of gamma/delta T lymphocytes in elderly: Implications for immunosenescence. Exp. Gerontol..

[112] Ligotti M.E., Accardi G., Aiello A., Calabrò A., Caruso C., Corsale A.M., Dieli F., Di Simone M., Meraviglia S., Candore G. (2024). Sicilian semi- and supercentenarians: Age-related Tγδ cell immunophenotype contributes to longevity trait definition. Clin. Exp. Immunol..

[113] Bulati M., Caruso C., Colonna-Romano G., Fulop T., Franceschi C., Hirokawa K., Pawelec G. (2019). B Cells in Centenarians and Their Offspring. Handbook of Immunosenescence.

[114] Frasca D., Diaz A., Romero M., Garcia D., Blomberg B.B. (2020). B Cell Immunosenescence. Annu. Rev. Cell Dev. Biol..

[115] Aiello A., Ligotti M.E., Cossarizza A., Caruso C. (2019). Centenarian Offspring as a Model of Successful Ageing. Centenarians.

[116] Castro J.P., Shindyapina A.V., Barbieri A., Ying K., Strelkova O.S., Paulo J.A., Tyshkovskiy A., Meinl R., Kerepesi C., Petrashen A.P. (2024). Age-associated clonal B cells drive B cell lymphoma in mice. Nat. Aging.

[117] Cancro M.P. (2020). Age-Associated B Cells. Annu. Rev. Immunol..

[118] Rubtsov A.V., Rubtsova K., Fischer A., Meehan R.T., Gillis J.Z., Kappler J.W., Marrack P. (2011). Toll-like receptor 7 (TLR7)-driven accumulation of a novel CD11c^+^ B-cell population is important for the development of autoimmunity. Blood.

[119] Mouat I.C., Horwitz M.S. (2022). Age-associated B cells in viral infection. PLoS Pathog..

[120] Rui K., Che N., Ma K., Zou H., Xiao F., Lu L. (2024). Coming of age: The formation and function of age-associated B cells. Cell Mol. Immunol..

[121] Edwards S.K., Desai A., Liu Y., Moore C.R., Xie P. (2014). Expression and function of a novel isoform of Sox5 in malignant B cells. Leuk. Res..

[122] Yang R., Avery D.T., Jackson K.J.L., Ogishi M., Benhsaien I., Du L. (2022). Human T-bet governs the generation of a distinct subset of CD11chighCD21low B cells. Sci. Immunol..

[123] Jackson T.A., Haga C.L., Ehrhardt G.R., Davis R.S., Cooper M.D. (2010). FcR-like 2 Inhibition of B cell receptor-mediated activation of B cells. J. Immunol..

[124] Dunn W.G., McLoughlin M.A., Vassiliou G.S. (2024). Clonal hematopoiesis and hematological malignancy. J. Clin. Investig..

[125] Deng Y., Peng Z., Ming K., Qiao X., Ye B., Liu Y., Wang H., Yang P., Zhang Y., Zhou K. (2025). Single-cell analysis of human thymus and peripheral blood unveils the dynamics of T cell development and aging. Nat. Aging.

[126] Hashimoto K., Kouno T., Ikawa T., Hayatsu N., Miyajima Y., Yabukami H., Terooatea T., Sasaki T., Suzuki T., Valentine M. (2019). Single-cell transcriptomics reveals expansion of cytotoxic CD4 T cells in supercentenarians. Proc. Natl. Acad. Sci. USA.

[127] Hashimoto K., Kojima-Ishiyama M., Inokuchi H., Tagami M., Sasaki T., Mizuguchi K., Okazaki Y., Taniuchi I., Ishigaki K., Hirose N. (2026). CD4 CTLs in supercentenarians: Signs of adaptive expansion in healthy aging. Cell Rep..

[128] Zhong J., Ding R., Jiang H., Li L., Wan J., Feng X., Chen M., Peng L., Li X., Lin J. (2023). Single-cell RNA sequencing reveals the molecular features of peripheral blood immune cells in children, adults and centenarians. Front. Immunol..

[129] Wang Y., Zhang Y., Gong G., Liu Q., Li L., Zhang M., Shen S., Wang R., Wu J., Xu W. (2025). Single-cell analysis of human peripheral blood reveals high immune response activity in successful ageing individuals. Mech. Ageing Dev..

[130] Karagiannis T.T., Dowrey T.W., Villacorta-Martin C., Montano M., Reed E., Belkina A.C., Andersen S.L., Perls T.T., Monti S., Murphy G.J. (2023). Multi-modal profiling of peripheral blood cells across the human lifespan reveals distinct immune cell signatures of aging and longevity. EBioMedicine.

[131] Dong C., Miao Y.R., Zhao R., Yang M., Guo A.Y., Xue Z.H., Li T., Zhang Q., Bao Y., Shen C. (2022). Single-Cell Transcriptomics Reveals Longevity Immune Remodeling Features Shared by Centenarians and Their Offspring. Adv. Sci..

[132] Trombetta C.M., Accardi G., Aiello A., Calabrò A., Caruso C., Ligotti M.E., Marchi S., Montomoli E., Neto M.M., Temperton N. (2024). Centenarians, semi and supercentenarians, COVID-19 and Spanish flu: A serological assessment to gain insight into the resilience of older centenarians to COVID-19. Immun. Ageing.

[133] Giri S., Batra L. (2025). Memory Cells in Infection and Autoimmunity: Mechanisms, Functions, and Therapeutic Implications. Vaccines.

[134] Jain A., Sturmlechner I., Weyand C.M., Goronzy J.J. (2023). Heterogeneity of memory T cells in aging. Front. Immunol..

[135] Kaech S.M., Cui W. (2012). Transcriptional control of effector and memory CD8^+^ T cell differentiation. Nat. Rev. Immunol..

[136] Larocca A.M.V., Bianchi F.P., Bozzi A., Tafuri S., Stefanizzi P., Germinario C.A. (2022). Long-Term Immunogenicity of Inactivated and Oral Polio Vaccines: An Italian Retrospective Cohort Study. Vaccines.

[137] Netea M.G., Schlitzer A., Placek K., Joosten L.A.B., Schultze J.L. (2019). Innate and Adaptive Immune Memory: An Evolutionary Continuum in the Host’s Response to Pathogens. Cell Host Microbe.

[138] O’Sullivan D. (2019). The metabolic spectrum of memory T cells. Immunol. Cell Biol..

[139] Pipkin M.E. (2011). Memories in the snow: Immune memory, persistent infection and chronic disease. EMBO Rep..

[140] Pollard A.J., Bijker E.M. (2021). A guide to vaccinology: From basic principles to new developments. Nat. Rev. Immunol..

[141] Ratajczak W., Niedźwiedzka-Rystwej P., Tokarz-Deptuła B., Deptuła W. (2018). Immunological memory cells. Cent. Eur. J. Immunol..

[142] Taub D.D., Ershler W.B., Janowski M., Artz A., Key M.L., McKelvey J., Muller D., Moss B., Ferrucci L., Duffey P.L. (2008). Immunity from smallpox vaccine persists for decades: A longitudinal study. Am. J. Med..

[143] De Biasi S., Lo Tartaro D., Rau M., Santacroce E., Paschalidis N., Bortoli F., Coppi F., Gibellini L., Rubio I., Cadore G. (2026). Smallpox-specific T cells in vaccinated centenarians exhibit different phenotypic and metabolic traits of long-term memory compared to SARS-CoV-2-specific memory T cells. Res. Sq..

[144] Ferrucci L., Fabbri E. (2018). Inflammageing: Chronic inflammation in ageing, cardiovascular disease, and frailty. Nat. Rev. Cardiol..

[145] Caiado F., Manz M.G. (2025). Clonal Hematopoiesis: Impact on Health and Disease. Hematol. Oncol..

[146] Pinti M., Gibellini L., Lo Tartaro D., De Biasi S., Nasi M., Borella R., Fidanza L., Neroni A., Troiano L., Franceschi C. (2023). A Comprehensive Analysis of Cytokine Network in Centenarians. Int. J. Mol. Sci..

[147] Zhou L., Ge M., Zhang Y., Wu X., Leng M., Gan C., Mou Y., Zhou J., Valencia C.A., Hao Q. (2022). Centenarians Alleviate Inflammaging by Changing the Ratio and Secretory Phenotypes of T Helper 17 and Regulatory T Cells. Front. Pharmacol..

[148] Plaza-Florido A., Alejo L.B., Pérez-Prieto I., Carrera-Bastos P., Muñoz M.R., Aldazabal I.P., Barranco-Gil D., Santos-Lozano A., Rodríguez-Romo G., Yanguas-Casás N. (2025). Even at 100+: Acute Exercise Modulates Inflammatory Pathways in Centenarians. Aging Cell.

[149] Accardi G., Calabrò A., Caldarella R., Caruso C., Ciaccio M., Di Simone M., Ligotti M.E., Meraviglia S., Zarcone R., Candore G. (2024). Immune-Inflammatory Response in Lifespan-What Role Does It Play in Extreme Longevity? A Sicilian Semi- and Supercentenarians Study. Biology.

[150] Accardi G., Bono F., Cammarata G., Aiello A., Herrero M.T., Alessandro R., Augello G., Carru C., Colomba P., Costa M.A. (2022). MiR-126-3p and miR-21-5p as Hallmarks of Bio-Positive Ageing; Correlation Analysis and Machine Learning Prediction in Young to Ultra-Centenarian Sicilian Population. Cells.

[151] Verlinden S.F. (2024). The genetic advantage of healthy centenarians: Unraveling the central role of NLRP3 in exceptional healthspan. Front. Aging.

[152] Lee G., Lee G.S. (2025). Potential Role of Inflammasomes in Aging. Int. J. Mol. Sci..

[153] Sizzano F., Collino S., Cominetti O., Monti D., Garagnani P., Ostan R., Pirazzini C., Bacalini M.G., Mari D., Passarino G. (2018). Evaluation of Lymphocyte Response to the Induced Oxidative Stress in a Cohort of Ageing Subjects, including Semisupercentenarians and Their Offspring. Mediat. Inflamm..

[154] Fries J.F. (2000). Compression of morbidity in the elderly. Vaccine.

[155] Kawas C.H. (2008). The oldest old and the 90+ Study. Alzheimer’s Dement..

[156] Biswas R., Kawas C., Montine T.J., Bukhari S.A., Jiang L., Corrada M.M. (2023). Superior Global Cognition in Oldest-Old Is Associated with Resistance to Neurodegenerative Pathologies: Results from The 90+ Study. J. Alzheimer’s Dis..

[157] Dominguez E.N., Corrada M.M., Kawas C.H., Stark C.E.L. (2024). Resilience to AD pathology in Top Cognitive Performers. Front. Aging Neurosci..

[158] Manoharan M.S., Lee G.C., Harper N., Meunier J.A., Restrepo M.I., Jimenez F., Karekatt S., Branum A.P., Gaitan A.A., Andampour K. (2025). The 15-Year Survival Advantage: Immune Resilience as a Salutogenic Force in Healthy Aging. Aging Cell.

[159] Ghosh R., Yousefzadeh M.J. (2025). TCF7-Linked Immune Resilience: A Salutogenic Biological Warranty for Longevity. Aging Cell.

[160] Fane M., Weeraratna A.T. (2020). How the ageing microenvironment influences tumour progression. Nat. Rev. Cancer.

[161] Solary E., Abou-Zeid N., Calvo F. (2022). Ageing and cancer: A research gap to fill. Mol. Oncol..

[162] Serrano M. (2016). Unraveling the links between cancer and aging. Carcinogenesis.

[163] Pavlidis N., Stanta G., Audisio R.A. (2012). Cancer prevalence and mortality in centenarians: A systematic review. Crit. Rev. Oncol. Hematol..

[164] Trastus L.A., D’Adda di Fagagna F. (2025). The complex interplay between aging and cancer. Nat. Aging.

[165] Harding C., Pompei F., Wilson R. (2012). Peak and decline in cancer incidence, mortality, and prevalence at old ages. Cancer.

[166] Harding C., Pompei F., Lee E.E., Wilson R. (2008). Cancer suppression at old age. Cancer Res..

[167] Miyaishi O., Ando F., Matsuzawa K., Kanawa R., Isobe K. (2000). Cancer incidence in old age. Mech. Ageing Dev..

[168] Franceschi C., Motta L., Motta M., Malaguarnera M., Capri M., Vasto S., Candore G., Caruso C. (2008). The extreme longevity: The state of the art in Italy. Exp. Gerontol..

[169] Salvioli S., Capri M., Bucci L., Lanni C., Racchi M., Uberti D., Memo M., Mari D., Govoni S., Franceschi C. (2009). Why do centenarians escape or postpone cancer? The role of IGF-1, inflammation and p53. Cancer Immunol. Immunother..

[170] Zarulli V., Barthold Jones J.A., Oksuzyan A., Lindahl-Jacobsen R., Christensen K., Vaupel J.W. (2018). Women live longer than men even during severe famines and epidemics. Proc. Natl. Acad. Sci. USA.

[171] Caruso C. (2025). Role of Sex and Gender in Ageing and Longevity.

[172] Marcon G., Tettamanti M., Capacci G., Fontanel G., Spanò M., Nobili A., Forloni G., Franceschi C. (2020). COVID-19 mortality in Lombardy: The vulnerability of the oldest old and the resilience of male centenarians. Aging.

[173] Couderc A.L., Correard F., Nouguerède E., Berbis J., Rey D., Daumas A., Villani P. (2021). Centenarians in nursing homes during the COVID-19 pandemic. Aging.

[174] Gellert P., Kohl R., Jürchott K., Hering C., Gangnus A., Steinhagen-Thiessen E., Kuhlmey A., Schwinger A. (2022). Centenarians From Long-Term Care Facilities and COVID-19-Relevant Hospital Admissions. J. Am. Med. Dir. Assoc..

[175] Cruces-Salguero S., Larrañaga I., García-Galindo A., Armañanzas R., Mar J., Matheu A. (2024). Analysis of response of centenarians of the Basque Country to COVID-19. J. Am. Geriatr. Soc..

[176] Espinosa O., Bejarano V., Franky I., Pagali S., Drummond M., Franco O.H. (2025). Mortality causes and health spending by gender and health conditions in octogenarians, nonagenarians and centenarians in Colombia. Sci. Rep..

[177] Aoki Y., Mehmet S.C. (2021). The COVID-19 pandemic appears to have increased longevity in Japanese centenarians. Age Ageing.

[178] Iwasaki A., Grubaugh N.D. (2020). Why does Japan have so few cases of COVID-19?. EMBO Mol. Med..

[179] Poulain M., Chambre D., Pes G.M. (2021). Centenarians exposed to the Spanish flu in their early life better survived to COVID-19. Aging.

[180] Caruso C., Accardi G., Aiello A., Calabrò A., Ligotti M.E., Candore G. (2023). Centenarians born before 1919 are resistant to COVID-19. Aging Clin. Exp. Res..

[181] de Castro M.V., Silva M.V.R., Naslavsky M.S., Scliar M.O., Nunes K., Passos-Bueno M.R., Castelli E.C., Magawa J.Y., Adami F.L., Moretti A.I.S. (2022). The oldest unvaccinated COVID-19 survivors in South America. Immun. Ageing.

